# The Abundance of Phenylalanine in the DNAJB6b Linker Governs Its Role in Amyloid Suppression

**DOI:** 10.1021/acsomega.6c07904

**Published:** 2026-10-05

**Authors:** Timas Merkelis, Erik Golovtchenko, Kalyani Sanagavarapu, Lovisa Björn, Cecilia Emanuelsson, Ulf Olsson, Sara Linse

**Affiliations:** † Division of Physical Chemistry, Department of Chemistry, 5193 Lund University, Naturvetarvägen 14, Lund 22100, Sweden; ‡ Biochemistry and Structural Biology, Department of Chemistry, 5193 Lund University, Naturvetarvägen 14, Lund 22100, Sweden

## Abstract

The molecular chaperone DNAJB6b inhibits the disease-related self-assembly of amyloid peptides in an efficient and ATP-independent manner. The molecular basis for this action is not yet fully understood, but the low-complexity region of DNAJB6b as a stand-alone peptide displays its antiamyloid formation properties and an ability to form self-assembled structures. Here, we studied the role of phenylalanine residues within the low-complexity region of the intact chaperone to gain insight into the role of these aromatic amino acid residues in chaperone-amyloid interactions. We designed, expressed, purified, and tested eight variants with various numbers of phenylalanine residues within the low-complexity region substituted with either alanine or serine. We show that substituting only 3 of these residues significantly affects the amyloid inhibition activity of the chaperone as well as its self-assembling ability, while substituting more residues affects these functions in a dose-dependent manner. Furthermore, we show that substituting all the phenylalanine residues within the low-complexity region reduces the amyloid inhibition activity of intact DNAJB6b to the equivalent of the isolated C-terminal domain, indicating that most of the chaperone activity stems from the aromatic residues within the low-complexity region.

## Graphical abstract

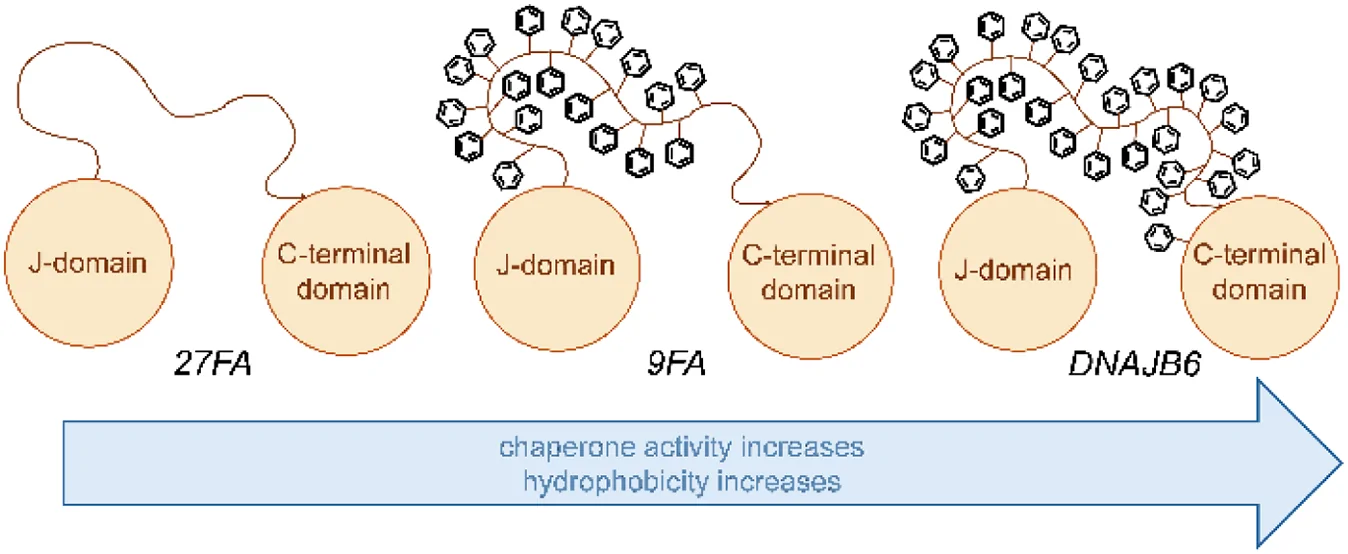

## Introduction

Molecular chaperones are essential regulators of protein homeostasis, facilitating correct protein folding and mitigating cellular damage arising from misfolded or aggregation-prone proteins.^1–3^ DNAJB6b (JB6) is a J-domain protein and a molecular chaperone from the Hsp40 family, widely expressed in the brain and muscles, is a potent amyloid aggregation suppressor.^4^ It has been associated with the prevention of aging-related neurodegenerative diseases and has been found to retard the aggregation of a number of amyloidogenic proteins, such as poly-Q-peptides,^5–7^ amyloid β peptides,^8,9^ tau,^10^ and α-synuclein.^11–13^ JB6 forms oligomeric aggregates in a concentration-dependent manner, above a critical concentration of around 100 nM;^14^ however, the monomeric subunit is the active form of the protein.^8^ While a mainly monomeric JB6 variant with part of the low complexity region deleted has been found to have reduced activity against protein aggregation,^15^ the self-assembly behavior may be a consequence of the same molecular features that govern the amyloid formation suppressing abilities.^4^

JB6 is made up of two globular domains, an N-terminal J-domain (JD), which is highly conserved throughout all DNAJ proteins and consists of four α helices, and a C-terminal domain (CTD), which contains four β strands. The two terminal domains are connected via a long low-complexity region (LCR), also referred to as the linker, that makes up approximately 45% of the residues of the full protein. The JD’s in DNAJ proteins bind to Hsp70 via a conserved HPD motif. The CTDs vary more within the J-domain proteins, and the CTD of JB6 is involved in amyloid-chaperone interactions.^16^ The LCR has been shown to play a role in the self-assembly and activity of the chaperone against amyloid formation.^7,15–18^ The GF rich region of the LCR also plays a role with-auto inhibition of binding to HSP70, with mutations within the GF motif destabilizing this property.^7,19^ The region also contains a short helix (residues 95–104), which is known to modulate this interaction with HSP70.^20,21^ JB6 has been recently shown to associate with FG-nucleoporins and prevent them from forming solid-like particles similar to amyloids, with the LCR playing a critical role.^22^ In a previous study,^17^ the 110-residue LCR (DNAJB6b 76–186) was expressed as a peptide devoid of the globular domains and was found to have amyloid suppression activity, demonstrating that it plays an important role in the overall chaperone activity.

The amino acid variation in the LCR is very limited,^17^ with over half of the residues being glycine (G), phenylalanine (F), serine (S), or threonine (T). It consists of two rather distinct halves referred to as the G/F rich and S/T rich segments,^15,18,23^ although both segments are G/F rich. Another J-domain chaperone of a similar function, DNAJB8, contains a similar LCR composed of G/F and S/T rich regions which are involved in its self-assembly behavior and interactions with amyloid proteins.^24^ Low complexity regions are found in several other proteins, where the promiscuity or fast dynamics in target recognition and condensate formation are important for their function. This includes the nuclear pore complex protein (which contains multiple F and G rich repeats^25^), and TAR DNA-binding protein 43 (TDP-43), containing low complexity, aromatic-rich segments, important for condensate formation and RNA- binding.^26,27^ Liquid–liquid phase separation and the co-condensation of intrinsically disordered proteins are also thought to mediate the formation of membrane-less organelles in cells.^28^

Phenylalanine is a highly hydrophobic amino acid residue with the fourth highest hydropathy index out of the proteogenic amino acids and has one of the highest transfer free energies from a hydrophobic environment to water.^29,30^ Two out of 64 codons code for F, and in proteins on average, it composes around 4% of the amino acid residues.^31^ However, in the LCR of JB6 it makes up no less than 24.5% of the residues (Figure 1), implying a key functional role of these F residues. Moreover, F has a high propensity toward β sheet formation^32^ and is less commonly found in disordered proteins.^32–34^ While it is often discussed as an important amino acid for protein π-π interactions^35^ and π stacking has been inferred to be important in tau fibril binding,^36^ simulations show an energetic preference for edge-to-face over face-to-face interactions of benzene rings^37,38^ and statistical analyses of protein core structures imply the same preference for phenylalanine rings.^39^

**Figure 1:**
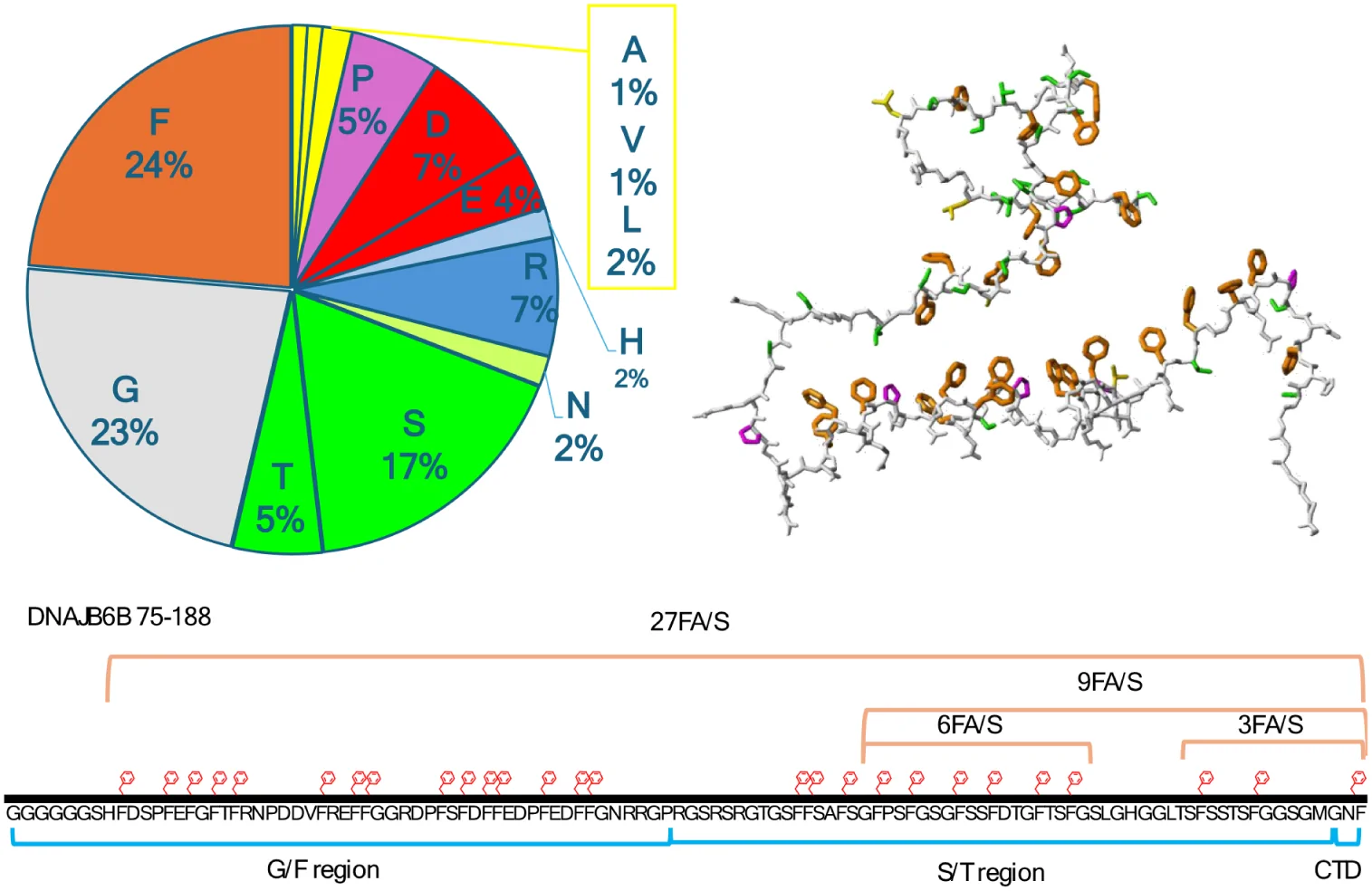
Amino acid residue composition of the JB6 LCR (left), an AlphaFold2 ^43^ illustration of the structure (right), and a representation of F substitution locations within the primary sequence (bottom). The same color coding is used in both panels, the right panel highlighting S, T, F, proline (P) and other hydrophobic residues.

There are 50 J-domain proteins in humans, 4 of which are DNAJA-proteins, 13 are DNAJB-proteins and the remaining are DNAJC-proteins. The DNAJA-proteins have an N-terminal J-domain and two CTDs, one of which includes a zinc-finger domain. The DNAJB-proteins have an N-terminal domain and either one or two CTDs; the DNAJC-proteins have the J-domain anywhere in the sequence.^40^ Of the DNAJB-proteins, only 5 are oligomeric and have a high abundance of ST-residues.^41^ These are referred to as DNAJBST-proteins. They have recently been shown by phylogenetic analyses to have evolved before the disease-causing amyloidogenic proteins.^42^ The closest homologue to DNAJB6 is DNAJB8, which is only expressed in testes. A DNAJB8 variant with some of the F residues of the S/T stretch replaced with S residues has been found to be mostly monomeric, but active against fibril formation.^24^ The S/T stretch of JB6 contains F residues in homologous positions, making these F residues of particular interest. According to Adupa et al. (2024),^21^ there are two GF-rich regions, GF1 and GF2, separated by D108, with GF2 discussed as having π-π interactions with the ST-region.

The present work investigates the importance of F residues within the LCR to JB6 self-assembly and its ability to suppress amyloid formation of client proteins. We have prepared eight JB6 variants (for sequences, see Supporting Information) in which F residues in the LCR were substituted with either alanine (A) or S, with either 3 (F175, 180, 188), 6 (F147, 150, 154, 157, 161, 164), 9 (combination of 3 + 6), or 27 substitutions (all F-residues in linker region). These substitutions proceed from the C-end toward the N-end within the LCR, with one substitution being present within the CTD. The 6FS variant of JB6 is analogous to DNAJB8_S3_, which was studied by Ryder *et. al* (2024),^24^ while the rest are meant to investigate the compounding effects of increasing amounts of F residue substitutions. Each variant was recombinantly expressed, purified, and characterized in terms of aggregation inhibition activity by thioflavin T (ThT) fluorescence, secondary structure by circular dichroism (CD) spectroscopy, and self-assembly behavior via mass photometry, size-exclusion chromatography (SEC), and flow-induced dispersion analysis (FIDA).

## Materials and Methods

### JB6 Expression and Purification

The JB6 variants were expressed in *E. coli* BL21­(DE3) pLysS Star through 15 h autoinduction and purified according to a previously published protocol,^44^ which includes removing acid-soluble contaminants by sonicating 5 times at pH 6.0, subsequently sonicating at pH 8.0 to disperse the chaperone into solution, passing through cation and anion exchange resins to remove charged contaminants, ammonium sulfate (AMS) precipitation and two rounds of size exclusion chromatography (SEC) in 1.6 M guanidinium hydrochloride (GuHCl) and a native buffer to remove large and small contaminants respectively, with the following modifications.

The 9FS variant required four additional rounds of sonication at pH 6.0 due to the formation of a particularly resistant cell pellet.

All variants, except 27FA and 27FS, were subjected to AMS precipitation between the two SEC steps. As a side note, in recent iterations we have replaced lyophilization with AMS precipitation between the SEC steps also for wild-type (WT) JB6.

The JB6 27FA and 27FS variants were not subjected to AMS precipitation at any stage and did not pass through the anion exchange resin (Q Sepharose Big Beads) at pH 8.0. The protein was instead bound to 50 mL of such resin and eluted with 50 mL of 2 M GuHCl. The eluate was subsequently lyophilized, resuspended in GuHCl, to a total concentration of roughly 8 M GuHCl, and immediately loaded onto a 16/600 mm Superose 6 column (GE Healthcare), equilibrated in 20 mM sodium phosphate, 0.2 mM EDTA, pH 8.

The identity of each purified variant was verified using mass spectroscopy in comparison with the theoretical masses based on their sequences (more in Supporting Information).

### Aβ42 Expression and Purification

The Aβ42 peptide used in this work, Aβ­(M1–42), was recombinantly expressed in *E. coli* BL21­(DE3) pLysS Star from a synthetic gene with *E. coli*-preferred codons,^45^ in LB medium with IPTG induction, and subsequently purified using ion exchange and size exclusion chromatography as previously described,^46^ and stored as lyophilized aliquots at −20 °C. Prior to the experiments, fresh monomeric Aβ42 was purified by size exclusion chromatography to ensure that no oligomers were present. Lyophilized aliquots were dissolved in 1 mL 6 M GuHCl, 20 mM sodium phosphate buffer (pH 8.0) before being injected into a 10/300 Superdex 75 increase column (Cytiva) with 20 mM sodium phosphate buffer 0.2 mM EDTA (pH 8.0) as the eluent at a flow rate of 0.5 mL/min. The peptide concentration was determined by integration of the absorbance of the peak at 280 nm of the collected fraction in the chromatogram using an extinction coefficient of 1440 M^–1^ cm^–1^.

### Aβ42 Aggregation via ThT Fluorescence

Fibril formation was monitored by following the ThT fluorescence in a FLUOstar plate reader (BMG LABTECH, Germany) with a 448 nm excitation filter and a 480 nm emission filter at 37 °C. PEGylated 96-well black polystyrene half a rea plates with a clear bottom (Corning 3881) were used, with no imposed shaking beyond the reading of the plate. Buffers were degassed and filtered using wwPTFE 0.2 μm 50 mm disc filters (Pall Corporation). ThT (CalBiochem) was dissolved in H_2_O, filtered (0.2 μm), and the concentration of the stock determined by absorbance of a diluted aliquot. In these experiments, the WT or variant JB6 were diluted from a high concentration stock (40–150 μM, depending on the sample), to 160 nM and further diluted with phosphate buffer to make a dilution series, then mixed with 8 μM Aβ42, 20 μM ThT in a 1:1 ratio to give final concentrations of 4 μM Aβ42 and 10 μM ThT.

### Circular Dichroism Spectroscopy

Circular dichroism (CD) spectra were recorded using a Jasco J-815 spectrometer with a Peltier thermostatic cell holder and Hellma Analytics QS high precision quartz cells with 4 mm path length at a scan rate of 10 nm/min, with 10 points/nm, slit width 1 nm, and a digital integration time of 16 s, averaging 5 scans. The protein concentrations of the samples used for CD spectroscopy were calculated from the absorbance spectrum in 2 M GuHCl in a 10 mm quartz cuvette using a Labbot instrument and an extinction coefficient of 14400 M^–1^ cm^–1^ at 280 nm (Probation Laboratories, Lund, Sweden).

### Size Exclusion Chromatography

Frozen or lyophilized aliquots of JB6 variants were prepared in 1 mL 2 M GuHCl, 20 mM sodium phosphate 0.2 mM EDTA (pH 8.0) at a concentration of 20 μM. This solution was injected into a Superose 6 column, and the absorbance was recorded at 280 nm. Twenty mM sodium phosphate buffer, 0.2 mM EDTA, (pH 8.0) was used as an eluent at a flow rate of 0.5 mL/min.

### Flow-Induced Dispersion Analysis

All FIDA measurements were performed using a FIDA NEO instrument from Fidabio with a fluorescence detector of an excitation range of 270–290 nm and an emission filter of 320–365 nm (Copenhagen, Denmark). Each JB6 sample was equilibrated at 10 μM in 20 mM phosphate buffer, 0.2 mM EDTA (pH 8.0) for 48 h at room temperature. The same buffer was used as the running buffer. Directly prior to measurement, Tween 20 was added to both the running buffer and the samples, resulting in a final concentration of 0.03% to reduce sample stickiness. The measurements were conducted in a 75 μm diameter 100 cm length modified glass capillary, permanently coated with linear polyacrylamide. Measurements were conducted at 25 °C, with each step described in Table 1 and repeated 3 times.

**Table 1:** Step-by-Step Experimental Procedure for FIDA Measurements

Component	Pressure (mbar)	Time (s)
0.1% phosphoric acid	3500	60
Milli-Q water	3500	60
Buffer	3500	60
JB6 sample	50	10
Buffer^a^	400	180
Milli-Q water	3500	60

a Measured component.

### Mass Photometry

All mass photometry measurements were performed using a Two MP instrument from Refeyn (Oxford, UK). All chaperone samples were equilibrated at 10 μM for 24 h at room temperature in 20 mM phosphate buffer, 0.2 mM EDTA (pH 8.0) prior to measurement. Each JB6 variant sample was also prepared at 100 nM in the same buffer. Directly before measurement each sample was diluted to 150 nM for JB6 wild type, and to 100 nM for the substituted chaperones.

## Results

In total, 8 JB6 variants were studied. The substitutions replaced either 3, 6, 9, or 27 F residues with either S or A residues starting from the CTD end. The activity of each of these variants as well as the self-assembly behavior and structure were compared with the WT JB6.

### Purification

While the purification essentially followed the protocol used for WT JB6, a few adjustments were necessary. The cell pellets of the variants were in general harder to fully break by sonication in comparison to WT. 3FA and 3FA pellets were the hardest ones to break and the 9FS mutant required 4 additional sonication rounds due to the pellet’s stickiness and gum-like consistency. In addition, variants 3FA, 6FA, 3FS and 6FS were more difficult to dissolve in 6 M GuHCl than WT before SEC. The 6FS variant formed a glass-like transparent layer on the centrifuge tube between the two SEC runs. Lastly, the ion exchange steps were modified for 27FA and 27FS variants due to binding to the Q Sepharose Big Beads resin and were eluted with 2 M GuHCl. These two variants also needed higher AMS concentrations to precipitate.

### Inhibition of Amyloid Formation

The activity of each JB6 variant against Aβ42 aggregation was measured at 37 °C by recording ThT fluorescence as a function of time at different JB6 concentrations in two repeat plates with 4 replicates of each concentration per plate. The overall delay in aggregation from each experiment is presented in Figure 2 in the form of the half time of aggregation, *t*_1/2_, defined as the point in time where the ThT fluorescence intensity is halfway in-between the initial baseline and the final plateau. The values obtained in the presence of chaperone were normalized by the *t*_1/2_ of Aβ42 alone within the same plate to give a delay factor. The Aβ42 concentration was 4.0 μM in all experiments. The JB6 concentration range from 5 to 80 nM represents a ratio of chaperone to Aβ42 from 1:800 to 1:50.

**Figure 2:**
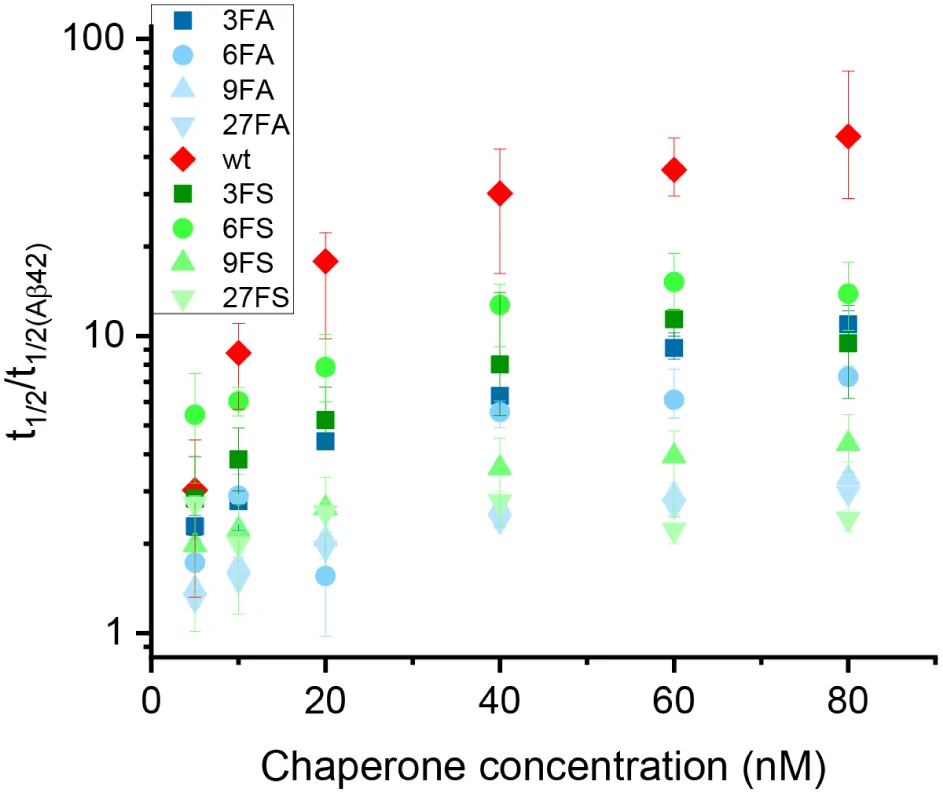
Effects on Aβ42 aggregation. The *t*_1/2_ of JB6 variant divided by the *t*_1/2_ of Aβ42 alone in the same plate, plotted versus the chaperone concentration on a logarithmic scale. The data points correspond to the average, with the error bars showing the deviation over multiple experiments. The concentration of Aβ42 is 4 μM in each experiment.

All variants display significantly less activity than WT JB6. In general, activity decreases, when more F residues are replaced in the LCR, WT being at least 4 times as active as any variant in terms of the concentration required to cause the same delay. The FA and FS variant counterparts displayed similar chaperone activity, with the S variants appearing to be overall slightly more active. The biggest difference was observed between variants 6FS and 6FA, with 6FS displaying a 2-fold increase in the delay of the aggregation compared to 6FA. The 27FA and 27FS variants show relatively low activity, delaying aggregation 3 times at 80 nM. The remaining activity of the two variants at 80 nM is similar to that of the CTD alone at 94 nM (Figure S32). The delay is slightly lower than that of CTD, likely due to a combination of lower concentration and an F substitution being present within the CTD. This substitution could impact the binding of CTD β strand 1 to Aβ42 and is related to Parkinson’s disease.^47^ It is likely important for dimerization of DNAJB6b as well.^48^

In another experiment, all variants were studied at 80 and 20 nM in the same plate for clearer comparison between variants, validating the above results. The 6FS variant was demonstrated to be the most active S variant against fibril formation, showing around the same activity as 3FA, as shown in Figure 3 and Figure S35.

**Figure 3:**
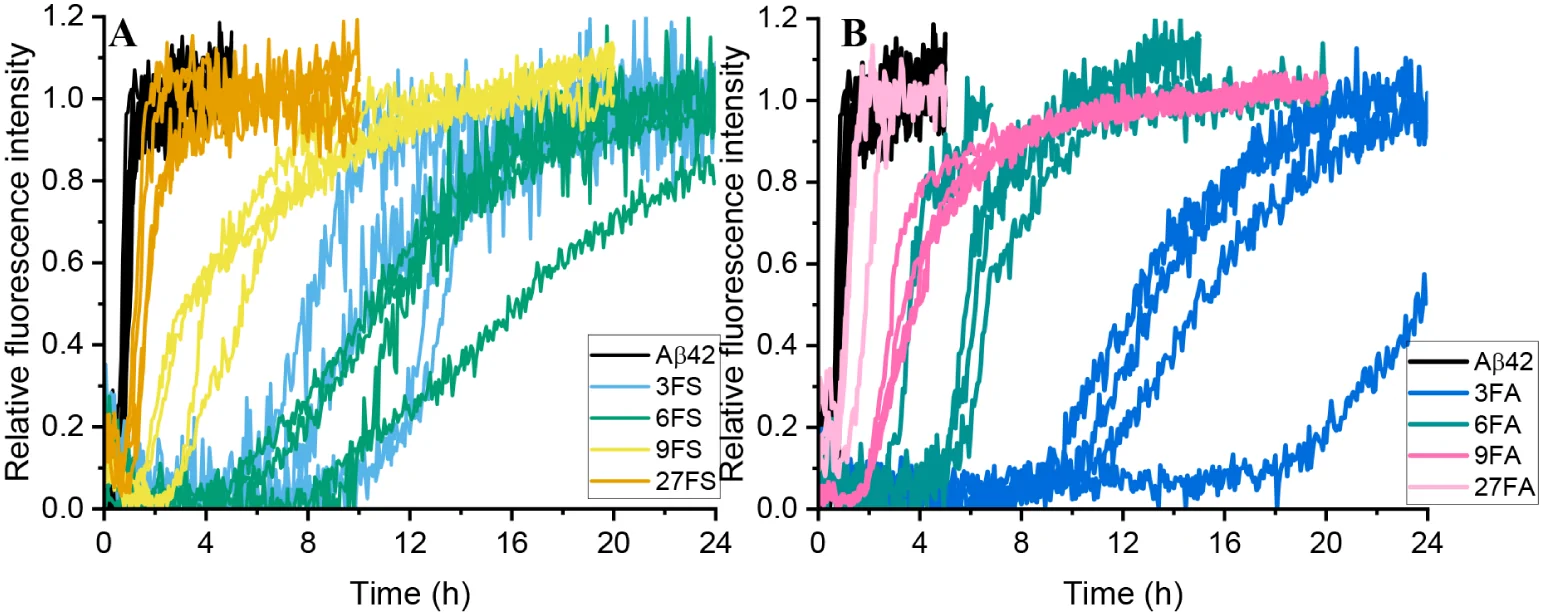
Effects on Aβ42 aggregation. ThT fluorescence versus time for all variants within the same plate at 80 nM. A- F to S variants. B- F to A variants. The concentration of Aβ42 is 4 μM.

### Self-Assembly

Each of the different variants was investigated and compared with WT JB6 using size exclusion chromatography (SEC), flow-induced dispersion analysis (FIDA), and mass photometry.

A sample of each variant of JB6 was prepared at 20 μM in 6 M GuHCl, 20 mM sodium phosphate buffer (pH 8.0) and 0.2 mM EDTA together with the WT. The samples were separately injected into a Superose 6 column to allow for more direct comparison between SEC runs of identical conditions. The samples were prepared in GuHCl for self-assembly during elution.

The results are presented in Figure 4. A significant amount of each variant (81–88%) was lost either in the filter or on the column due to attractive resin-protein interactions, losing more of the serine variants than alanine variants. Under the experimental conditions, each variant and the wild type are allowed to aggregate on the column. However, only the wild type self-assembles, while the variants exhibit no self-assembly on the column, possibly due to self-assembly within the variants taking longer than the elution time. The variants coelute with the WT monomer, while the WT self-assembled structures elute first. The equilibrium between wild type monomers and oligomers is reached at a time scale greater than the experiment time, allowing oligomers and monomers to separate on the column, resulting in the two-state elution. The F27S and F27A variants elute slightly earlier, even though they have lower mass and display no self-assembly when measured with other methods, meaning this is likely due to weaker interactions with the resin. This also leads to less material being lost on the column. This apparent higher mass was also observed on a Coomassie-stained SDS PAGE gel, where the apparent mass of variants increased with the number of substitutions. This change in apparent mass is sometimes observed on SDS PAGE gels.^49^ This result indicates that the substitution variants are less oligomeric than WT or adsorb to the resin more strongly.

**Figure 4:**
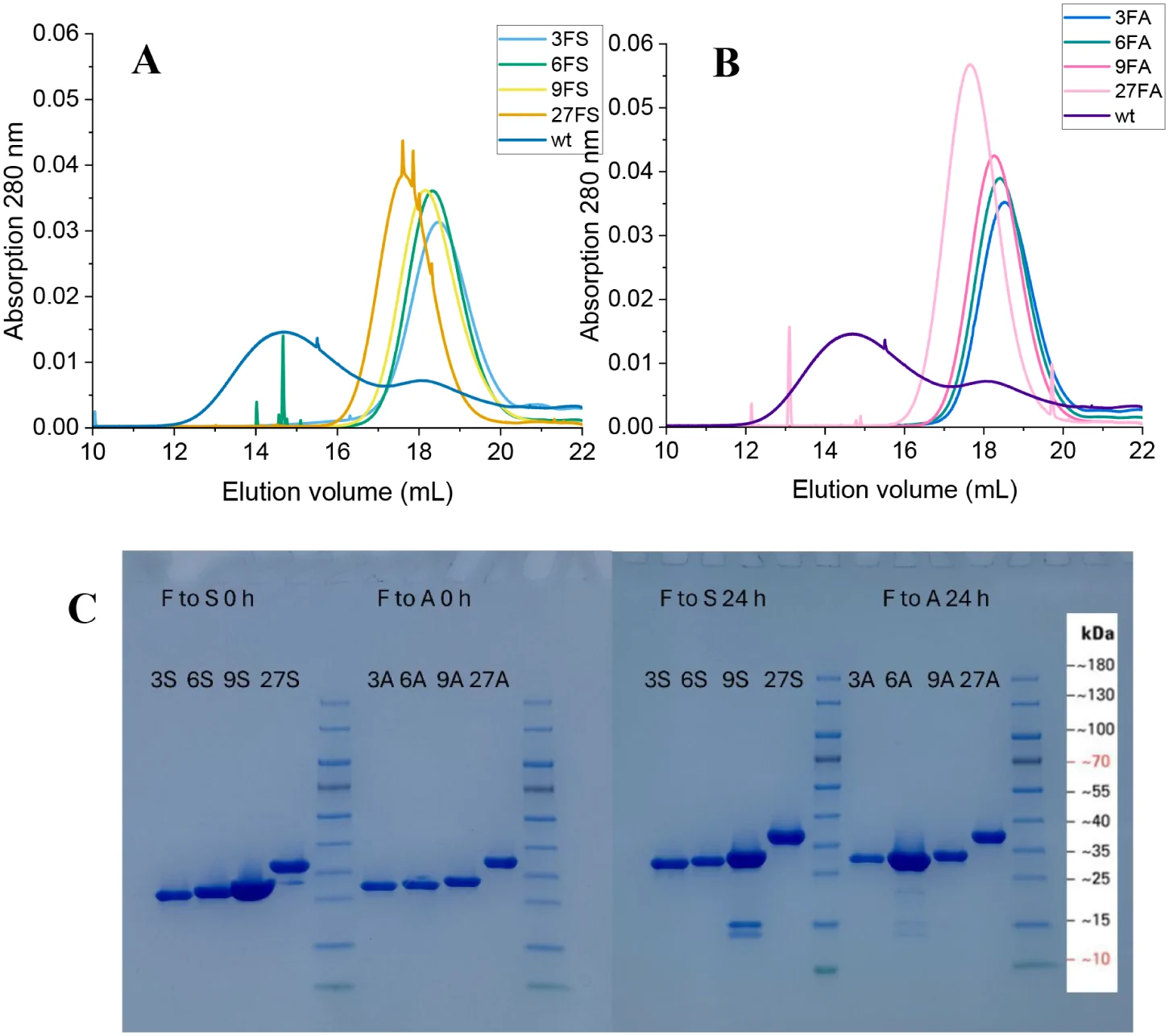
Size exclusion chromatograms of each variant injected at 20 μM in 0.8 mL 6 M GuHCl on a 25 mL Superose 6 column. The path length through the detector was 5 mm for each variant and 3 mm for the WT. A) The F to S substituted variants, B) F to A substituted variants. C) Coomassie-stained 4–20% SDS PAGE gels (Biorad) with tris/glycine as a running buffer of JB6 variant aliquots directly after thawing (left) and after incubation at 37 °C for 24 h (right). Variant aliquots were prepared at the order of 10 μM. Each well was loaded with 7.5 μL of aliquots and mixed with 2.5 μL of 4× Laemmli buffer. Wells are labeled according to the substitutions.

FIDA was used to measure hydrodynamic radii of JB6 variants. The data is displayed in Figure 5. The intrinsic fluorescence data was fitted with increasing numbers of Gaussian components until the fit satisfied the constraints of being R^2^ > 0.999 with the individual populations contributing to at least 0.5% of the summed signal. Due to peak asymmetry, only the first half of each Taylorgram was fitted.

**Figure 5:**
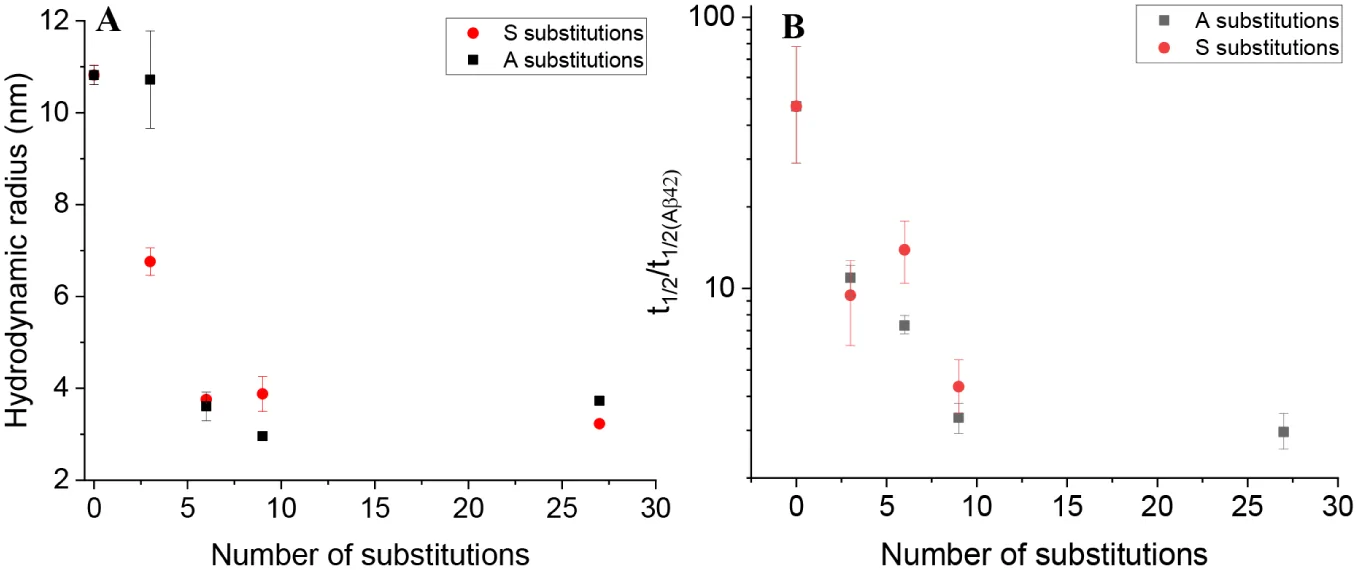
Hydrodynamic radii evaluated by FIDA. A) Average hydrodynamic radius of JB6 variants versus the number of substitutions from F to A (black squares) or F to S (red circles). B) *t*_1/2_ of each variant at 80 nM (a ratio of chaperone to Aβ42 of 1:40) from ThT fluorescence experiments. F to A variant data is represented by black squares, while F to S data by red circles. The data points for 27FS and 27FA overlap.

The fitted Gaussian components were used to calculate the diffusion coefficient, *D*, from the variance, σ^2^,according to Poulsen et al. (2015):^50^

1 $$D=\frac{a^{2}}{24\sigma^{2}}t_{R}$$

where *a* is the capillary radius, and *t*_*R*_ is the peak appearance time. The hydrodynamic radius, *R*_*H*_, is then obtained from diffusion coefficient using the Stokes–Einstein equation.

2 $$R_{H}=\frac{k_{B}T}{6\pi \eta D}$$

Here, *k*_*B*_ is Boltzmann’s constant, *T* is the absolute temperature, and η is the solvent viscosity, approximated to be that of water at 25 °C (0.89 mPa·s).

All JB6 variants, apart from 3FA, could be fitted by one species of a dominant population according to the constraints. 3FA JB6 can be represented by two species of roughly equal populations, one larger and one smaller. *R*_H_ of the species versus the number of substitutions is presented in Figure 5 (only the species of a larger size is shown for 3FA). The chaperones appear to be increasingly less prone to self-assembly with more F residues substituted, as seen by the reduction in the hydrodynamic radius. While FIDA gives insights to the general self-assembly behavior, we are unable to determine the distribution of aggregation numbers from this data, since it does not describe how many subunits are present in each species. Additionally, the presence of detergent may impact the oligomerization of JB6. Self-assembly behavior was therefore also investigated by mass photometry.

Data from mass photometry was also obtained for the substitutional variants and compared to WT JB6. The results are presented in Figure 6. Each sample was equilibrated at 10 μM at room temperature for 24 h and then rapidly diluted to 100 nM just prior to the measurement. In the mass photometry experiment, the occurrences of particles of different masses were counted over a time period of 2 min. This measurement time is short compared to the micellar dissociation time of WT JB6 ^51^ and likely for the substituted variant dissociation times as well. Therefore, the observed size distributions are expected to represent the equilibrium size distribution at 10 μM to a good approximation. A second sample of each variant was left to equilibrate for 24 h at 100 nM and not diluted after the equilibration. Only the WT and 3*A*/3S variants exhibited measurable self-assembly behavior. The average aggregation number was 31 for the WT JB6, 14 for the 3FA variant, and 13 for the 3FS variant. The samples prepared at 100 nM displayed only a monomer peak, with too little signal for oligomers to allow for meaningful analysis (Figure S39).

**Figure 6:**
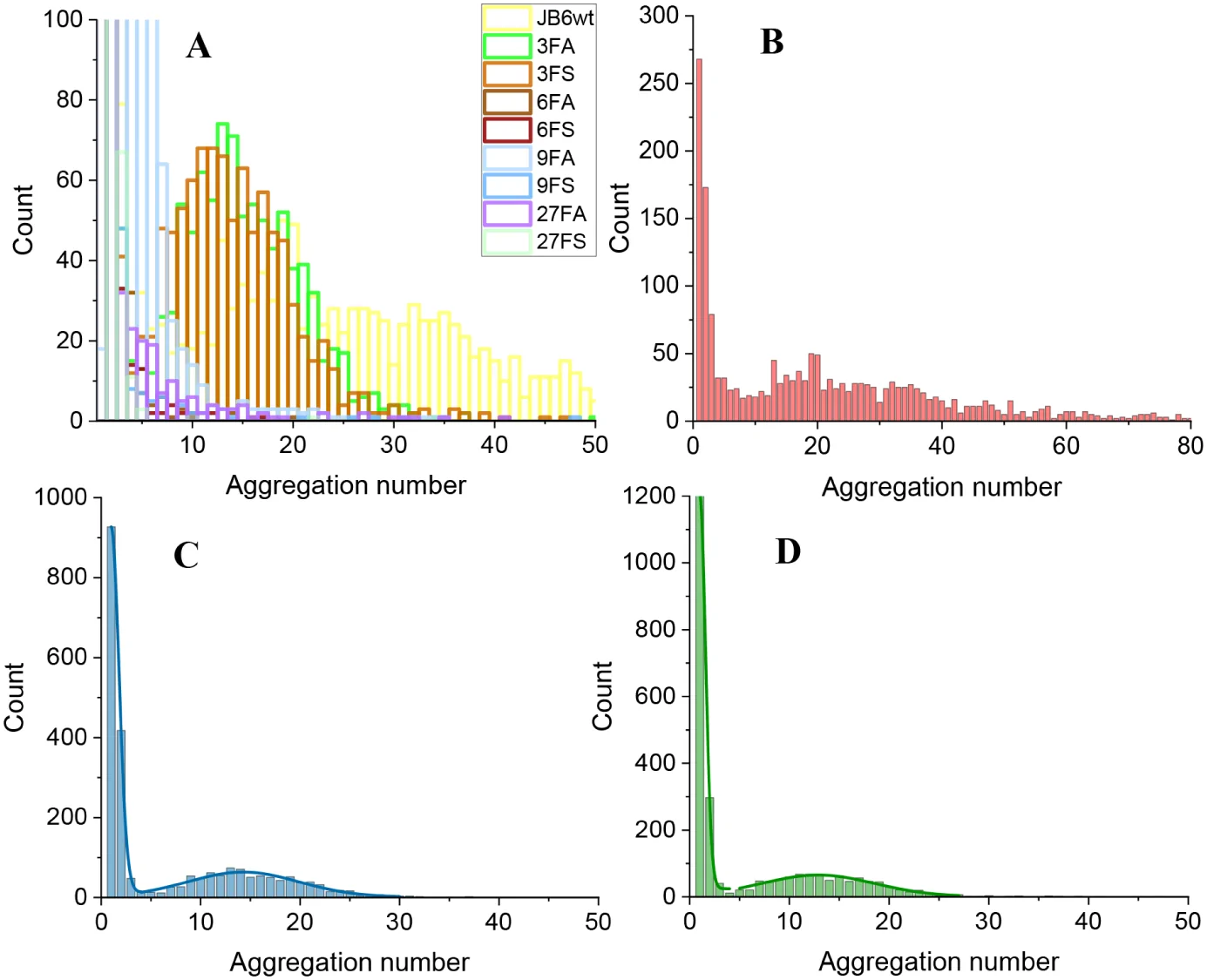
Mass photometry data for each JB6 variant (A), the WT JB6 (B), 3FA (C), and 3FS (D).

### CD Spectroscopy

To further evaluate the structural effects of the substituted variants CD-spectra of each variant were recorded at 2 μM in a 4 mm cuvette and compared to JB6 WT. The results are presented in Figure 7. Based on the spectra, all F substitutions seem to introduce secondary structure changes. A loss of absolute spectral intensity can be seen with the 3FS, 6FS, and 9FS variants, while an increase in α-helical signal observed as a broad or double minimum in the spectrum can be seen with 3FA, 6FA, and especially 9FA compared to the WT. The F to S substitutions lead to dramatic signal losses, which may be interpreted as the protein being more disordered. This change might be a result of a reduction of secondary structure in the mostly disordered LCR compared to WT. The F to A substitutions may thus possibly induce the formation of more helix in the LCR, which is not unexpected given that alanine is a known α-helix-promoting residue.^52^

**Figure 7:**
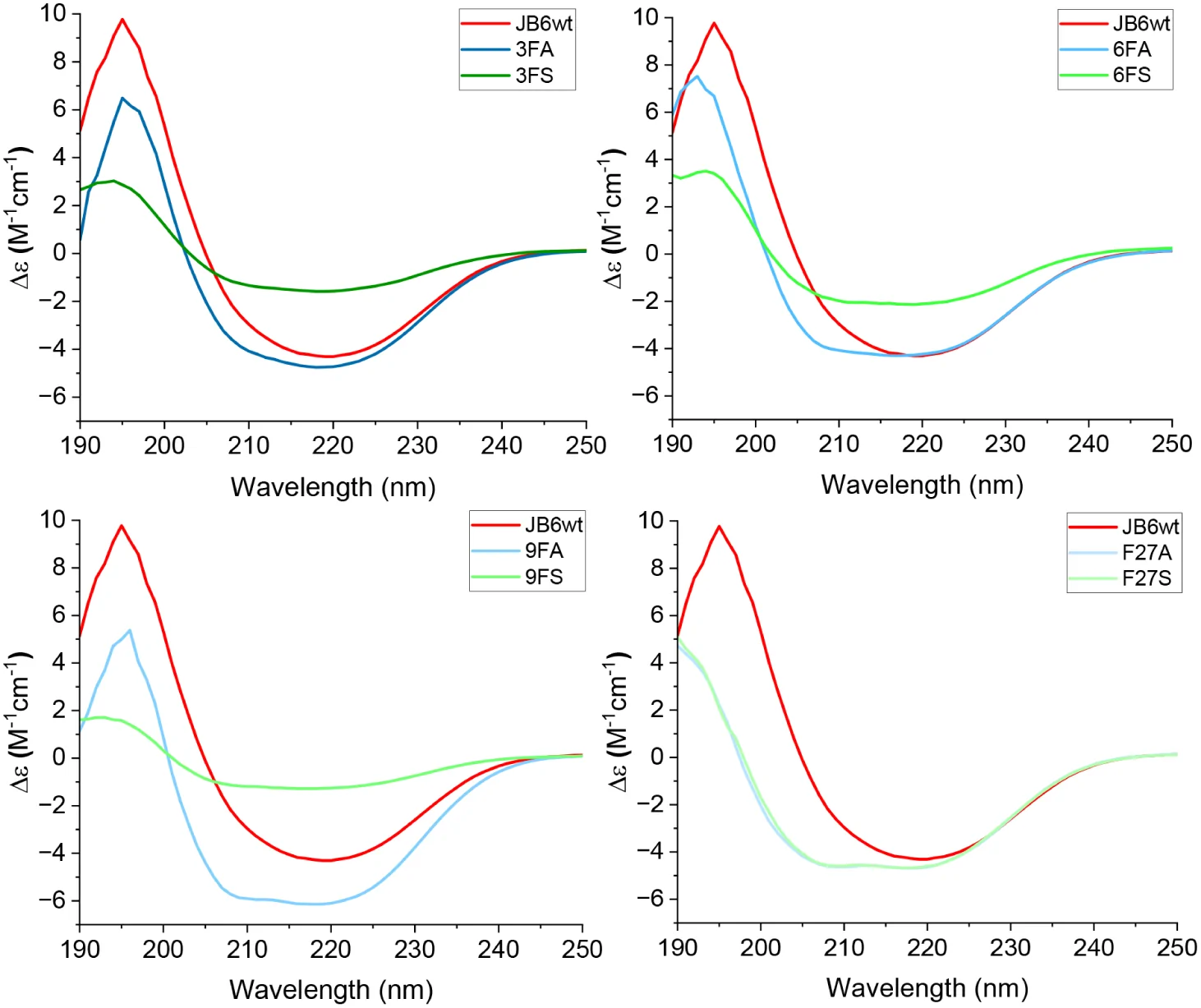
CD spectra for each variant and the wild type of JB6 show an increased α-helix signal for A-substituted variants and a loss of secondary structure in S-substituted variants. Both A and S variants with all F residues in the LCR substituted show analogous secondary structure signals.

The two variants with all F residues substituted to A or S seem to have a similar structure, which differs from the other variants, observed as a broader double minimum in the 205–230 nm range. This indicates a significant structural change upon the substitution of all the F-residues in the LCR.^7^

## Discussion

The results presented show that all F residue substitutions of JB6 significantly impact both its activity as an amyloid suppressor and its self-assembly. The variants with all 27 F residues in the LCR substituted displayed essentially no activity (apart from CTD contributions) or self-assembly at 10 μM, indicating that F residues are indeed critical for the antiamyloid properties of the DNAJB6 chaperone.

The effects are dose-dependent, with each additional set of three substitutions further reducing both properties of the chaperone. The variant 6FS remained more active than the other variants, in spite of being structurally affected and essentially monomeric. These features resemble the findings in Ryder et al. (2024),^24^ in which the same region in the closest homologue, DNAJB8, was substituted in the corresponding region and the variant was found to be monomeric and active against fibrillation. This region is referred to as GF2 in Adupa et al. (2024),^21^ and was discussed as having π-π interactions with F side chains in the ST-region.

### The Role of the Composition of the LCR

The LCR of JB6 consists of only a small number of amino acid residues, with over 50% being, in order of abundance, G, S, D, and P, all commonly found in low-complexity regions. Another 24% is F, which is often depleted in such regions.^32–34^ The LCR is highly hydrophobic due to the abundance of F residues, which due to a lack of secondary structure within the region are more likely to attractively interact with amyloid proteins in the JB6 monomer. Most of the region is likely structured via its primary sequence to prevent folding, keeping the F residues exposed and the region dynamically available to interact with various amyloid forming proteins. This property can also explain the drive toward self-assembly. Highly hydrophobic residues are exposed to water, resulting in a high chemical potential of the chaperone. Forming micelle-like structures effectively hides the hydrophobic residues from water, which lowers the free energy of the system compared to a higher concentration of free monomers.

### The Role of Serine versus Alanine

The S substituted variants of JB6 generally showed slightly higher activity against fibril formation than their A substituted counterparts. This could be explained by the changes in secondary structure of the chaperones as observed by CD spectroscopy (Figure 7).^53^ While the A variants displayed an increase in helical signal, S variants displayed an increased random coil signal. The S variants may thus have left the LCR unstructured, while in the A variants the LCR has gained more secondary structure. In the case of the S variants, this could leave the remaining F residues more available to interact with Aβ42. The 27FA and 27FS variants showed no significant differences, as all F residues in the LCR were substituted.

### On the Amphiphilicity of JB6

In previous work, we discussed the role of the globular JB6 domains in increasing chaperone solubility.^17^ The LCR devoid of globular domains is less soluble and prefers to phase separate from the water phase, while the domains limit the aggregation, resulting in small oligomeric assemblies. While the CTD has been shown to affect amyloid formation, it seems to target secondary nucleation almost exclusively and is much less potent in this function than the full protein, which targets both primary and secondary nucleation processes.^16^ The CTD is about as active in amyloid inhibition as the 27FA/S variants, suggesting that the remaining activity in these variants can be ascribed to the CTD. This not only shows that the F residues within the LCR are crucial for chaperone-amyloid interactions, but also that the role of globular domains in these interactions is comparatively small. While the CTD contributes to the interactions with amyloids, the function of the domains is more important to self-assembly and interaction with other chaperone proteins in the case of the JD.

Other variants of JB6 with substitutions or deletions within the LCR have been studied. Variants with S/T residues substituted or removed have been shown to have reduced ability to inhibit polyQ aggregation,^54^ and Aβ42 aggregation,^55^ as well as a loss in self-assembly.^56^ The loss in activity was discussed to arise either from reduced hydrogen bonding capacity or increased hydrophobicity in the region.^54^

Our results in this work show that decreasing hydrophobicity and thereby increasing the solubility of JB6 by introducing substitutions affects both self-assembly and the activity against fibril formation. This loss in activity could be explained by a less hydrophobic LCR being less able to attractively interact with highly hydrophobic amyloid oligomers. On the other hand, making the chaperone more hydrophobic, such as when the domains are removed, causes the chaperone to become too insoluble and aggregate. It appears that an optimal trade off requires keeping the chaperone “unhappy”,^4^ resulting in the formation of small oligomers while not increasing the chemical potential of the protein to the point of phase separation.

## Conclusions

The substitution of F residues in the JB6 LCR reduces both the ability of suppressing amyloid aggregation and self-assembly in a dose-dependent manner. Antiamyloid formation activity and self-assembly are significantly lowered even with the introduction of 3 substitutions. The F residues therefore are key to both functions, and almost all 27 are needed to keep them, likely due to the hydrophobic/aromatic character of the residues driving chaperone-amyloid interactions. These residues exist in a primarily unstructured region. A lack of secondary structure drives the oligomeric self-assembly, making the residues less exposed to water. While there are differences between A and S substitutions, they are not as impactful to chaperone activity against amyloid formation as an increased number of substitutions.

## Supplementary Material



## Notes

List of variant primary sequences, mass spectra, purification procedure data, additional ThT kinetic curves, absorbance spectra of the variants, FIDA curves, and omitted mass photometry data (PDF)
