# Supplementary material for: The Abundance of Phenylalanine in the DNAJB6b Linker Governs Its Role in Amyloid Suppression
Source: ACS Omega. 2026 Oct 5;11(41):63724–33. doi: 10.1021/acsomega.6c07904 (PMC13689251; doi:10.1021/acsomega.6c07904)
Supplement: Supplementary file 1 [file ao6c07904_si_001.pdf]

The abundance of phenylalanine in the DNAJB6b linker governs its role in amyloid suppression

Timas Merkelis*[1], Erik Golovtchenko[2], Kalyani Sanagavarapu[2], Lovisa Björn[2], Cecilia Emanuelsson[2], Ulf Olsson[1], Sara Linse[2]

[1]Division of Physical Chemistry, Department of Chemistry, Lund University, Lund, Naturvetarvägen 14, 22100, Sweden and [2]Biochemistry and Structural Biology, Department of Chemistry, Lund University, Lund, Naturvetarvägen 14, 22100, Sweden

*timas.merkelis@chem.lu.se

Supporting information

Variant sequences

1) JB6-3FS (F175S, F180S, F188S)

MVDYYEVLGVQRHASPEDIKKAYRKLALKWHPDKNPENKEEAERKFKQVAEAYEV
LSDAKKRDIYDKYGKEGLNGGGGGGSHFDSPFEFGFTFRNPDDVFREFFGGRDPFSF
DFFEDPFEDFFGNRRGPRGSRSRGTGSFFSAFSGFPSFGSGFSSFDTGFTSFGSLGHGG
LTSSSSTSSGGSGMGNSKSISTSTKMVNGRKITTKRIVENGQERVEVEEDGQLKSLTIN
GKEQLLRLDNK

2) JB6-3FA (F175A, F180A, F188A)

MVDYYEVLGVQRHASPEDIKKAYRKLALKWHPDKNPENKEEAERKFKQVAEAYEV
LSDAKKRDIYDKYGKEGLNGGGGGGSHFDSPFEFGFTFRNPDDVFREFFGGRDPFSF
DFFEDPFEDFFGNRRGPRGSRSRGTGSFFSAFSGFPSFGSGFSSFDTGFTSFGSLGHGG
LTSASSTSAGGSGMGNAKSISTSTKMVNGRKITTKRIVENGQERVEVEEDGQLKSLTI
NGKEQLLRLDNK

3) JB6-6FS (F147S, F150S, F154S, F157S, F161S, F164S)

MVDYYEVLGVQRHASPEDIKKAYRKLALKWHPDKNPENKEEAERKFKQVAEAYEV
LSDAKKRDIYDKYGKEGLNGGGGGGSHFDSPFEFGFTFRNPDDVFREFFGGRDPFSF
DFFEDPFEDFFGNRRGPRGSRSRGTGSFFSAFSGSPSSGSGSSSSDTGSTSSGSLGHGG
LTSFSSTSFGGSGMGNFKSISTSTKMVNGRKITTKRIVENGQERVEVEEDGQLKSLTIN
GKEQLLRLDNK

4) JB6-6FA (F147A, F150A, F154A, F157A, F161A, F164A)

MVDYYEVLGVQRHASPEDIKKAYRKLALKWHPDKNPENKEEAERKFKQVAEAYEV
LSDAKKRDIYDKYGKEGLNGGGGGGSHFDSPFEFGFTFRNPDDVFREFFGGRDPFSF
DFFEDPFEDFFGNRRGPRGSRSRGTGSFFSAFSGAPSAGSGASSADTGATSAGSLGHG
GLTSFSSTSFGGSGMGNFKSISTSTKMVNGRKITTKRIVENGQERVEVEEDGQLKSLTI
NGKEQLLRLDNK

5) JB6-9FS as a combination of variants 1 and 3

MVDYYEVLGVQRHASPEDIKKAYRKLALKWHPDKNPENKEEAERKFKQVAEAYEV
LSDAKKRDIYDKYGKEGLNGGGGGGSHFDSPFEFGFTFRNPDDVFREFFGGRDPFSF
DFFEDPFEDFFGNRRGPRGSRSRGTGSFFSAFSGSPSSGSGSSSSDTGSTSSGSLGHGG
LTSSSSTSSGGSGMGNSKSISTSTKMVNGRKITTKRIVENGQERVEVEEDGQLKSLTIN
GKEQLLRLDNK

6) JB6-9FA as a combination of variants 2 and 4

MVDYYEVLGVQRHASPEDIKKAYRKLALKWHPDKNPENKEEAERKFKQVAEAYEV
LSDAKKRDIYDKYGKEGLNGGGGGGSHFDSPFEFGFTFRNPDDVFREFFGGRDPFSF
DFFEDPFEDFFGNRRGPRGSRSRGTGSFFSAFSGAPSAGSGASSADTGATSAGSLGHG
GLTSASSTSAGGSGMGNAKSISTSTKMVNGRKITTKRIVENGQERVEVEEDGQLKSLT
INGKEQLLRLDNK

7) JB6-27FS – all F to S in LCR

MVDYYEVLGVQRHASPEDIKKAYRKLALKWHPDKNPENKEEAERKFKQVAEAYEV
LSDAKKRDIYDKYGKEGLNGGGGGGSHSDSPSESGSTSRNPDDVSRESSGGRDPSSS
DSSEDPSEDSSGNRRGPRGSRSRGTGSSSSASSGSPSSGSGSSSSDTGSTSSGSLGHGG
LTSSSSTSSGGSGMGNSKSISTSTKMVNGRKITTKRIVENGQERVEVEEDGQLKSLTIN
GKEQLLRLDNK

8) JB6-27FA – all F to A in LCR

MVDYYEVLGVQRHASPEDIKKAYRKLALKWHPDKNPENKEEAERKFKQVAEAYEV
LSDAKKRDIYDKYGKEGLNGGGGGGSHADSPAEAGATARNPDDVAREAAGGRDPAS
ADAAEDPAEDAAGNRRGPRGSRSRGTGSAASAASGAPSAGSGASSADTGATSAGSL
GHGGLTSASSTSAGGSGMGNAKSISTSTKMVNGRKITTKRIVENGQERVEVEEDGQL
KSLTINGKEQLLRLDNK

Mass Spectroscopy

Digestion of protein samples

Proteins were digested in solution as follows; The pH of the samples were adjusted to 7.8 by adding ammonium bicarbonate (ABC) to a final concentration of 100 mM. The proteins were reduced by addition of DL-Dithiothreitol (DTT) (Sigma-Aldrich) in 50 mM ABC to a final concentration of 5 mM and incubated in 37°C for 30 min, followed by alkylation using iodoacetamide (IAA, Sigma-Aldrich) at a final concentration of 12 mM and incubation in the

dark for 20 min. Finally Chymotrypsin (Promega, Madison, WI, USA) was added to a ratio or 1:100 (w/w) protease:protein, and the samples were digested overnight at 37 °C. The next day formic acid (FA) was added to a final concentration of 0.5 %. The peptides were then cleaned up by C18 reversed-phase micro columns using an 2% Acetonitrile (ACN), 0.1% FA equilibration buffer and an 80% ACN, 0.1% FA elution buffer. Finally, the collected samples were dried in a fume hood and resuspended in 15 µl 2% ACN, 0.1% FA.

LC-MSMS

The samples were then injected to an ultra-high pressure nanoflow chromatography system (nanoElute, Bruker Daltonics). The peptides were loaded onto an Acclaim PepMap C18 (5 mm, 300 µm id, 5 µm particle diameter, 100 Å pore size) trap column (Thermo Fisher Scientific) and separated on a Bruker Pepsep Ten C18 (75 µm × 10 cm, 1.9 µm particle size) analytical column (Bruker Daltonics). Mobile phase A (2% ACN, 0.1% FA) was used with the mobile phase B (0.1% FA in ACN) for 45 min to create a gradient (from 2 to 17% B in 20 min, from 17 to 34% B in 10 min, from 34 to 95% B in 3 min, at 95%B for 12 min) at a flow rate of 300 nl/min and a column oven temperature of 50 ◦C. The peptides were analysed on a quadrupole time-of-flight mass spectrometer (timsTOF Pro, Bruker Daltonics), via a nano electrospray ion source (Captive Spray Source, Bruker Daltonics) in positive mode, controlled by the OtofControl 5.1 software (Bruker Daltonics). The temperature of the ion transfer capillary was 180°. A DDA method was used to select precursor ions for fragmentation with one TIMS-MS scan and 10 PASEF MS/MS scans. The TIMS-MS scan was acquired between 0.60–1.6 V s/cm^2 and 100–1700 m/z with a ramp time of 100 ms. The 10 PASEF scans contained maximum of 10 MS/MS scans per PASEF scan with a collision energy of 10 eV. Precursors with maximum 5 charges with intensity threshold to 5000 a. u. and a dynamic exclusion of 0.4 s were used.

Data analysis

Raw data were processed using Mascot Distiller (version 2.8) and searched in Mascot Daemon (version 2.8.0) against a Database with DNAJB6 sequences containing the mutations. The following settings were used; precursor ion tolerance 10 ppm, MS/MS fragment mass tolerance 0.015 Da, None as protease, Oxidation (M), Acetylation (K) and Deamidation (NQ) as variable modification and Carbamidomethylation (C) as fixed modification.

JB6_3FA

Query	Start – End	Observed	Mr(expt)	Mr(calc)	ppm	M	Score	Expect	Rank	U	Peptide
6970	173 – 189	735.8325	1469.6505	1469.6416	6.03	0	99	1.4e-10	1	U	L.TSASSTSAGGSGMGNAK.S

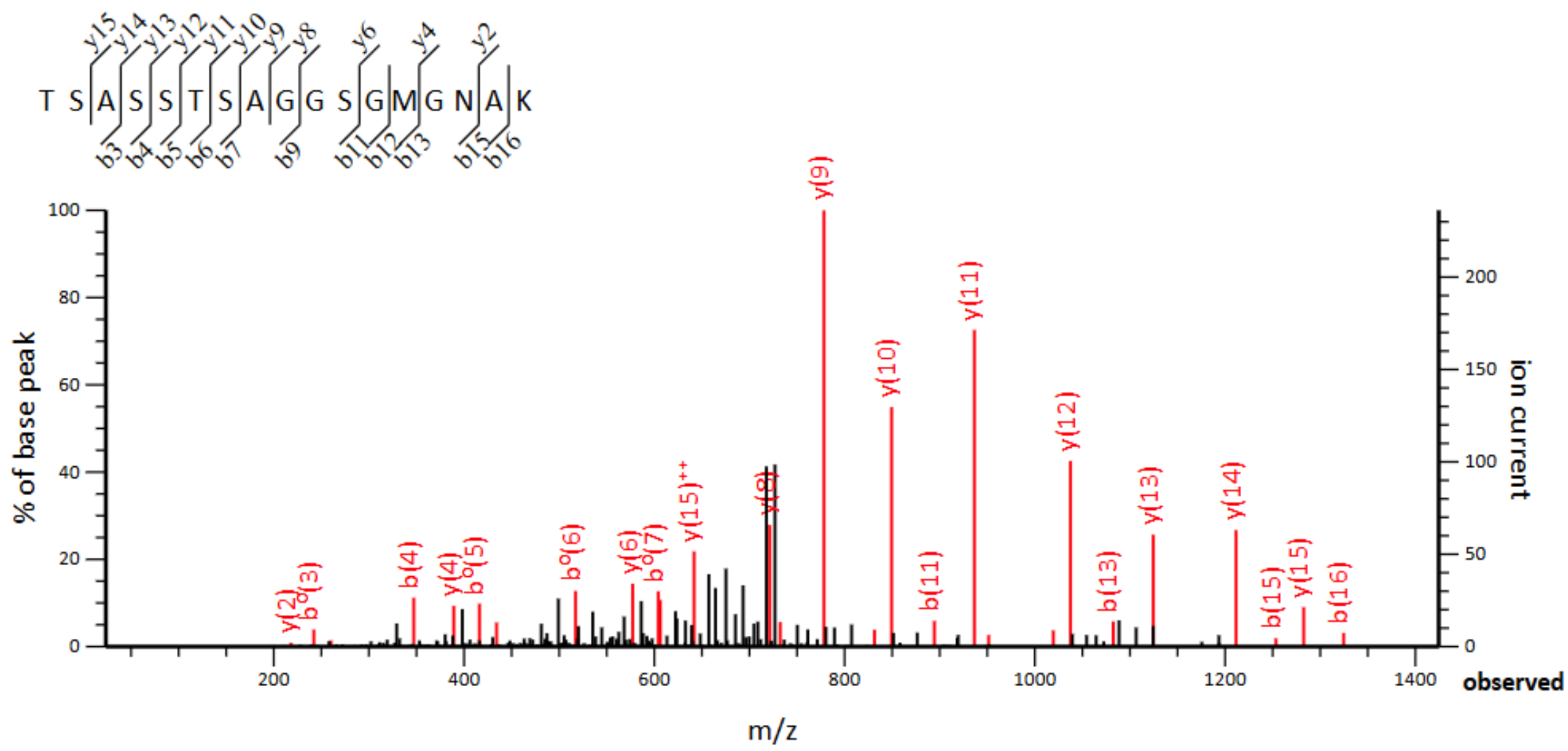

Figure S1. Mass spectrum of the 3FA variant.

JB6_3FS

9434 173 – 191 859.8659 1717.7173 1717.7425 -14.6 0 129 2.4e-13 1 L.TSSSSTSSGGSGMGNSKSI.S

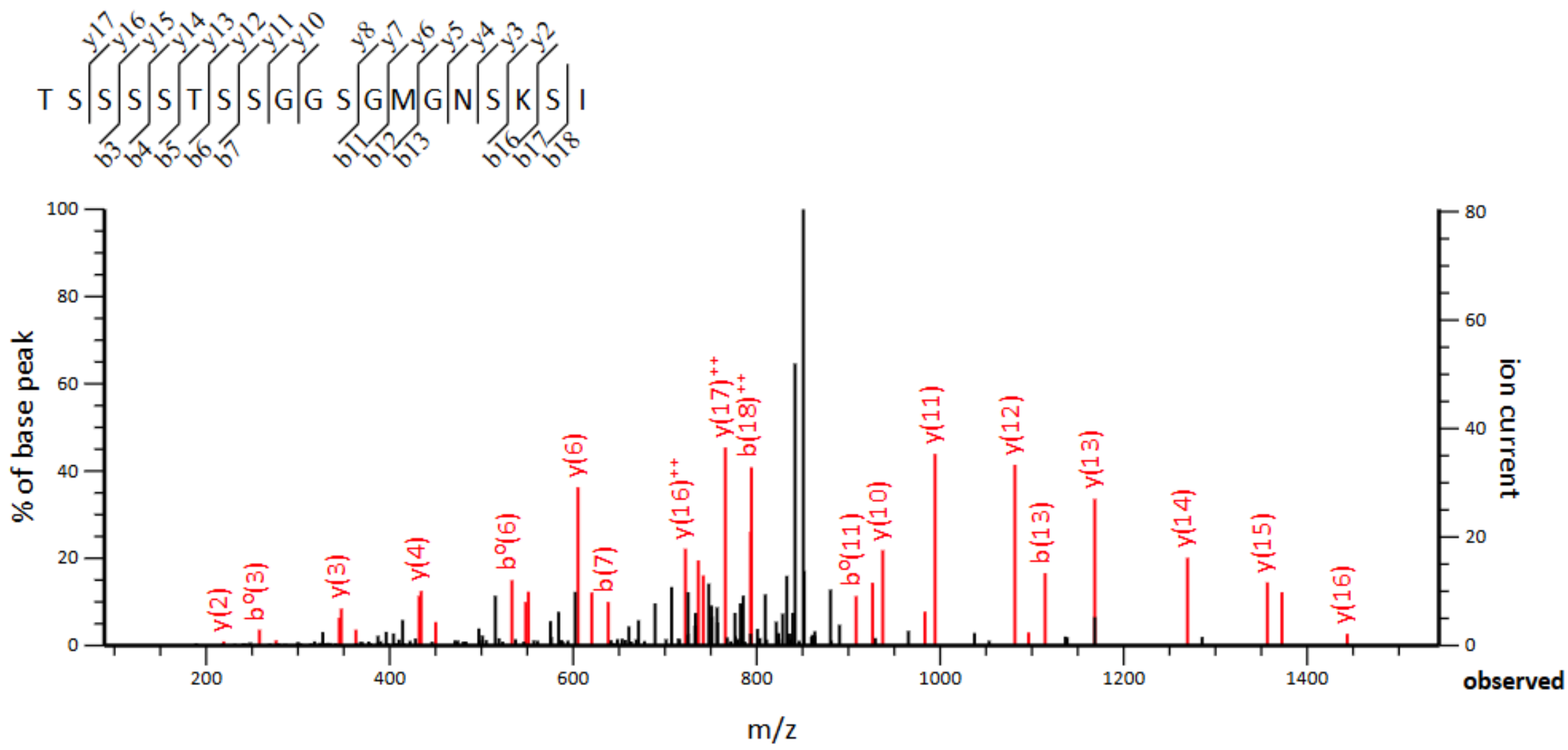

Figure S2. Mass spectrum of the 3FS variant.

JB6_6FA

9740 144 – 167 1007.4538 2012.8930 2012.892 0.36 0 92 6e-10 1 U A.FSGAPSAGSGASSADTGATSAGSL.G

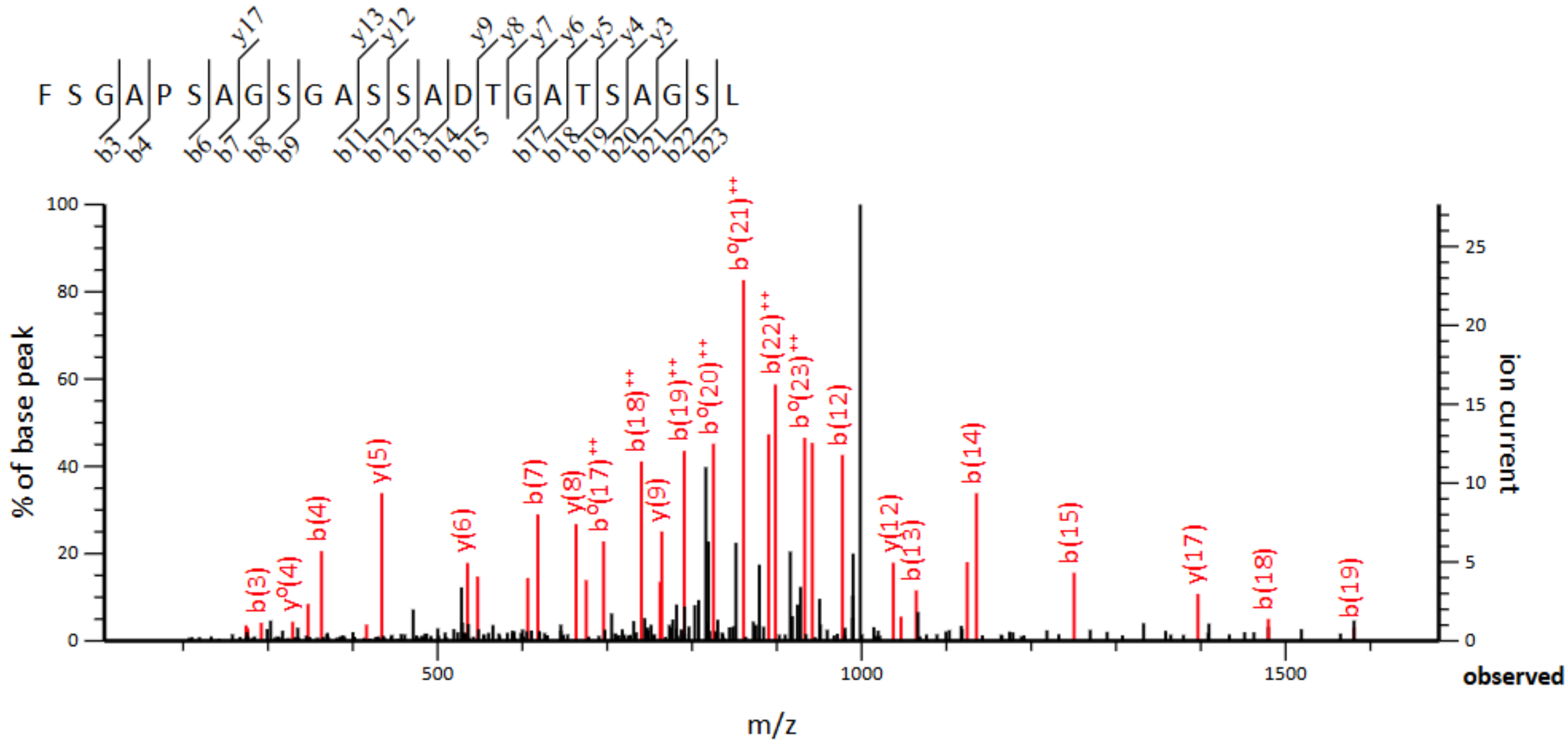

Figure S3. Mass spectrum of the 6FA variant.

JB6_6FS

8068 145 – 167 981.8993 1961.7840 1961.7934 -4.79 0 175 2.9e-18 1 F.SGSPSSGSGSSSSDTGSTSSGSL.G

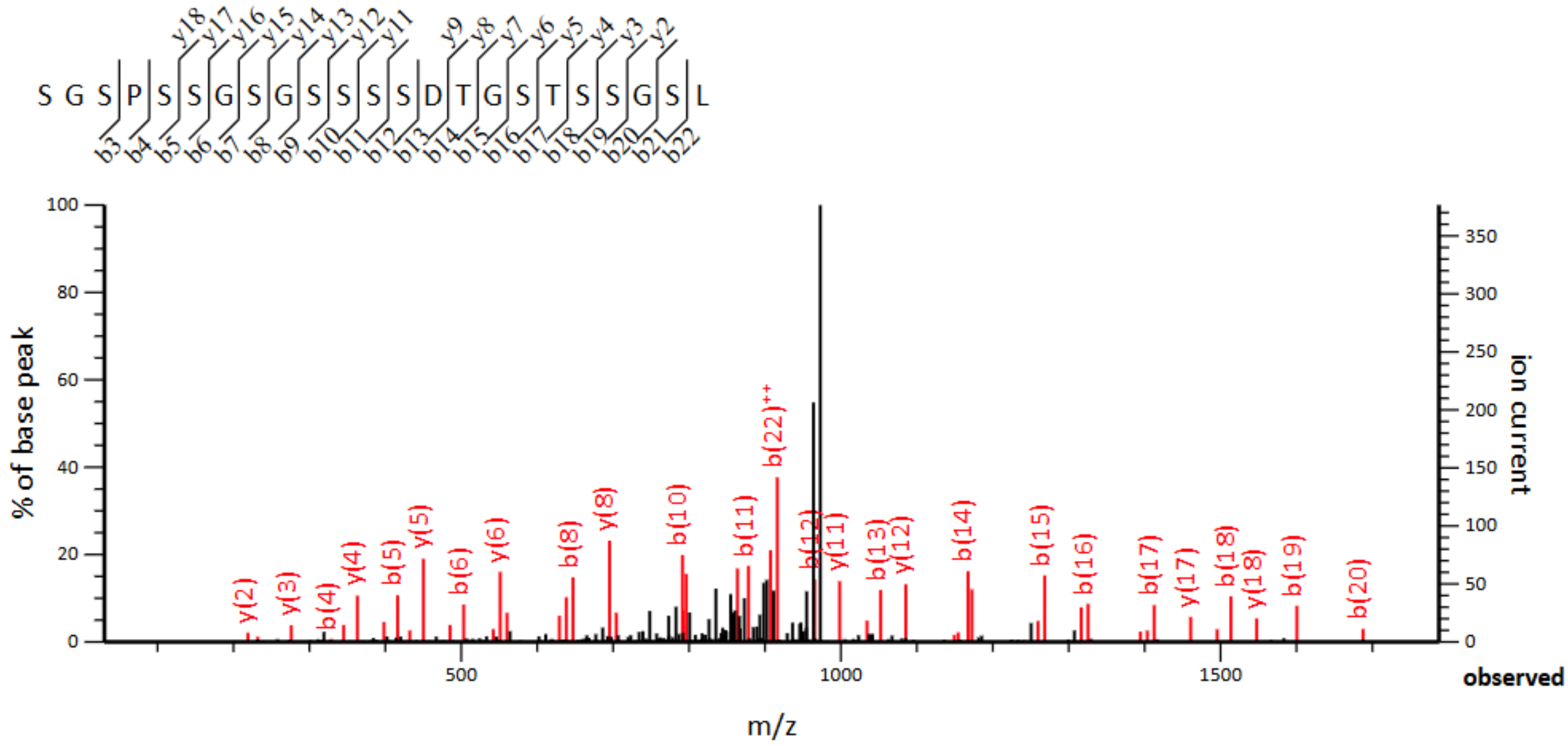

Figure S4. Mass spectrum of the 6FS variant.

JB6_9FA

18815 145 – 173 797.0350 2388.0831 2388.0790 1.75 0 118 2.2e-12 1 F.SGAPSAGSGASSADTGATSAGSLGHGGLTS

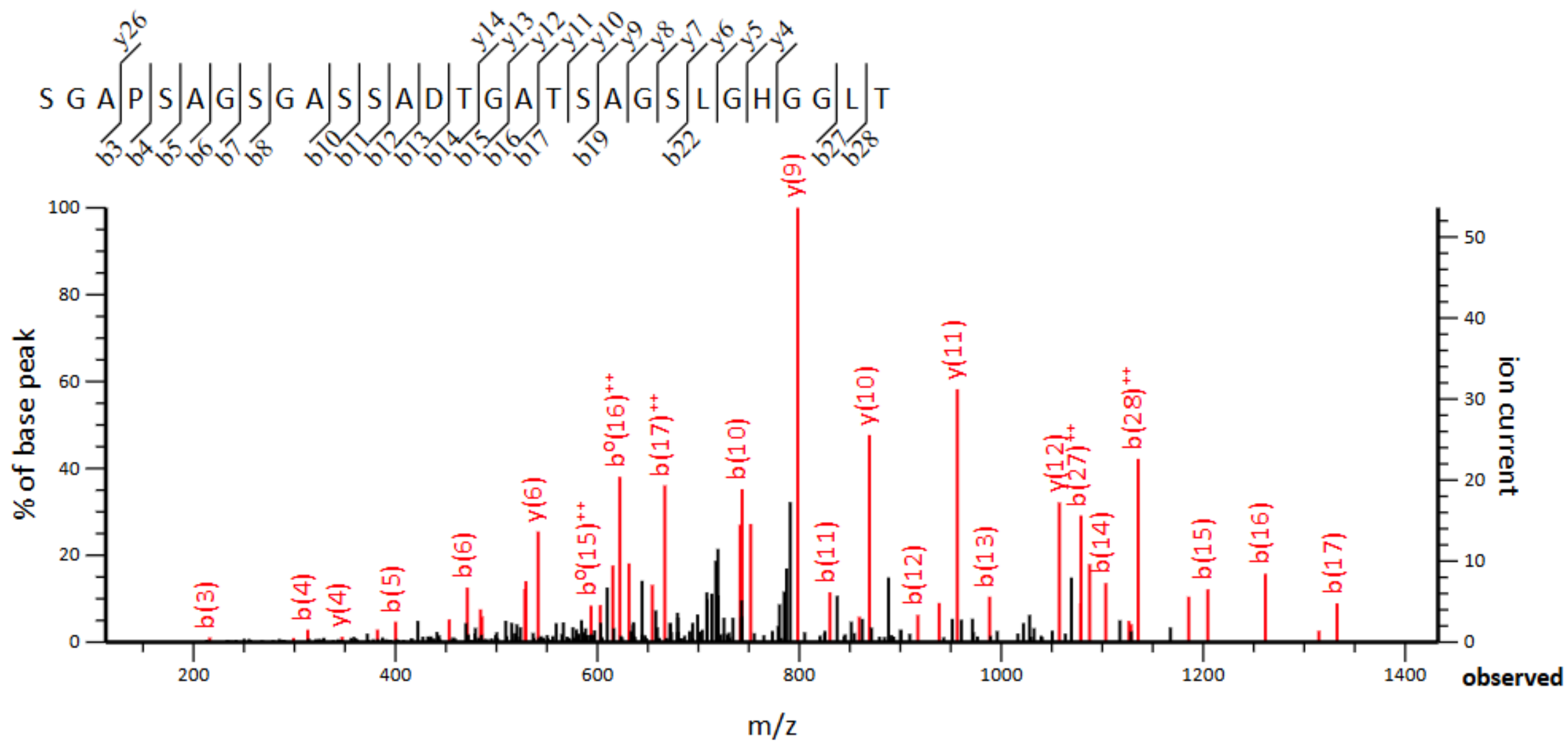

Figure S5. Mass spectrum of the 9FA variant.

6233 173 – 189 735.8285 1469.6424 1469.6416 0.50 0 92 5.7e-10 1 U L.TSASSTSAGGSGMGNAKS

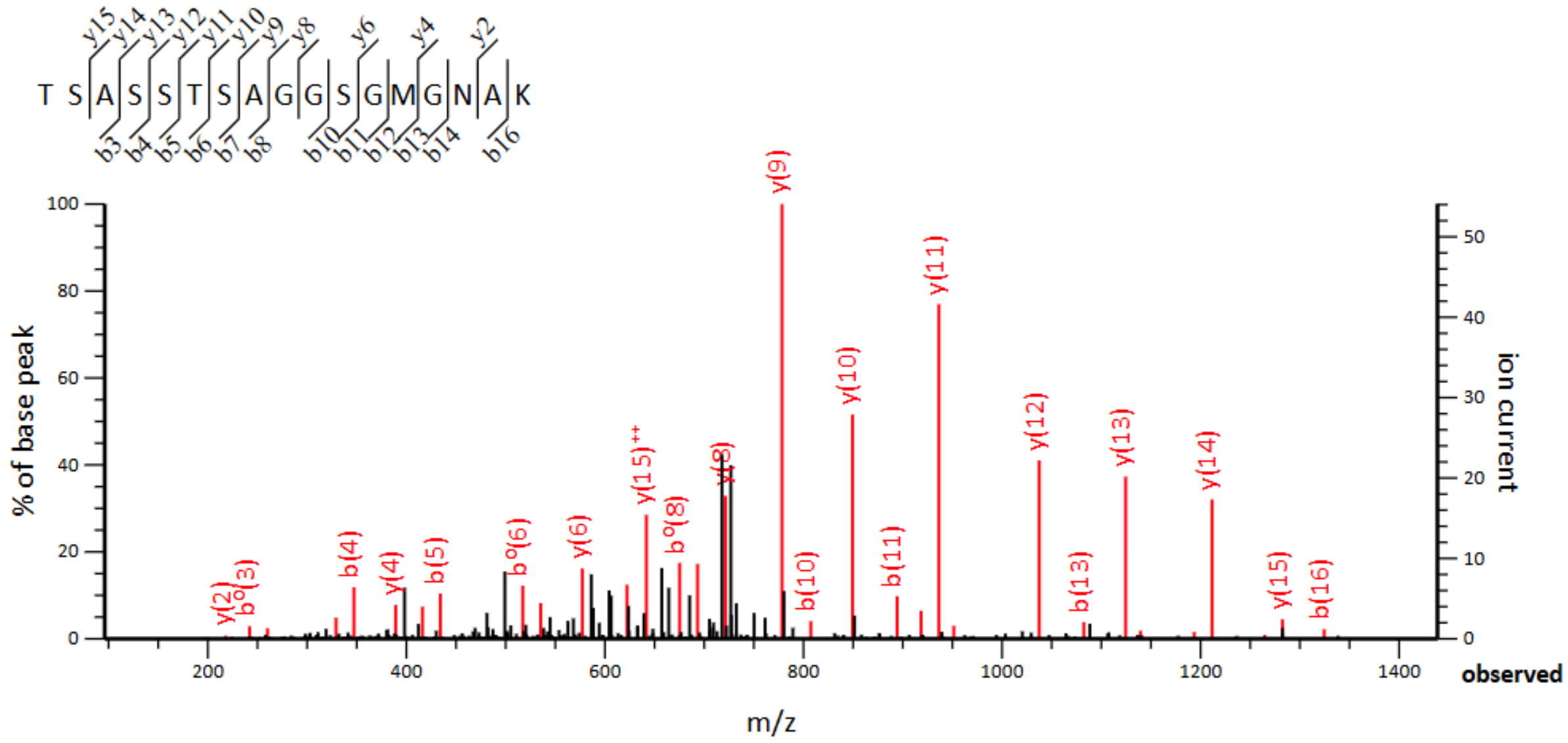

Figure S6. Mass spectrum of the 9FA variant (repeat).

JB6_9FS

6099 145 – 167 981.8993 1961.7840 1961.7934 -4.78 0 169 1.3e-17 1 F.SGSPSSGSGSSSSDTGSTSSGSL.G

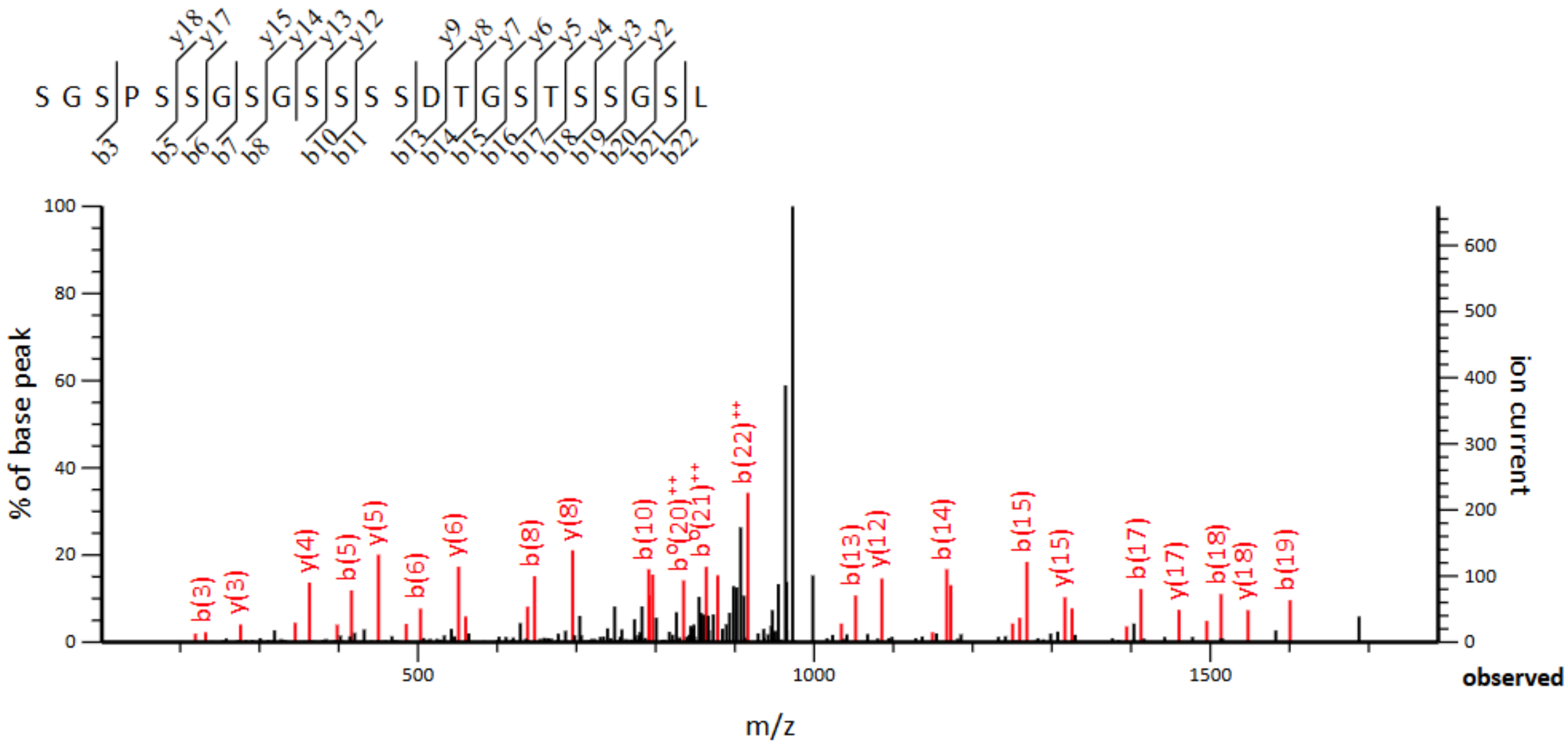

Figure S7. Mass spectrum of the 9FS variant.

4790 173 – 191 860.3644 1718.7142 1718.7265 -7.14 0 107 2.3e-11 1 L.TSSSSTSSGGSGMGNSKSI.S
+ Deamidated (NQ)

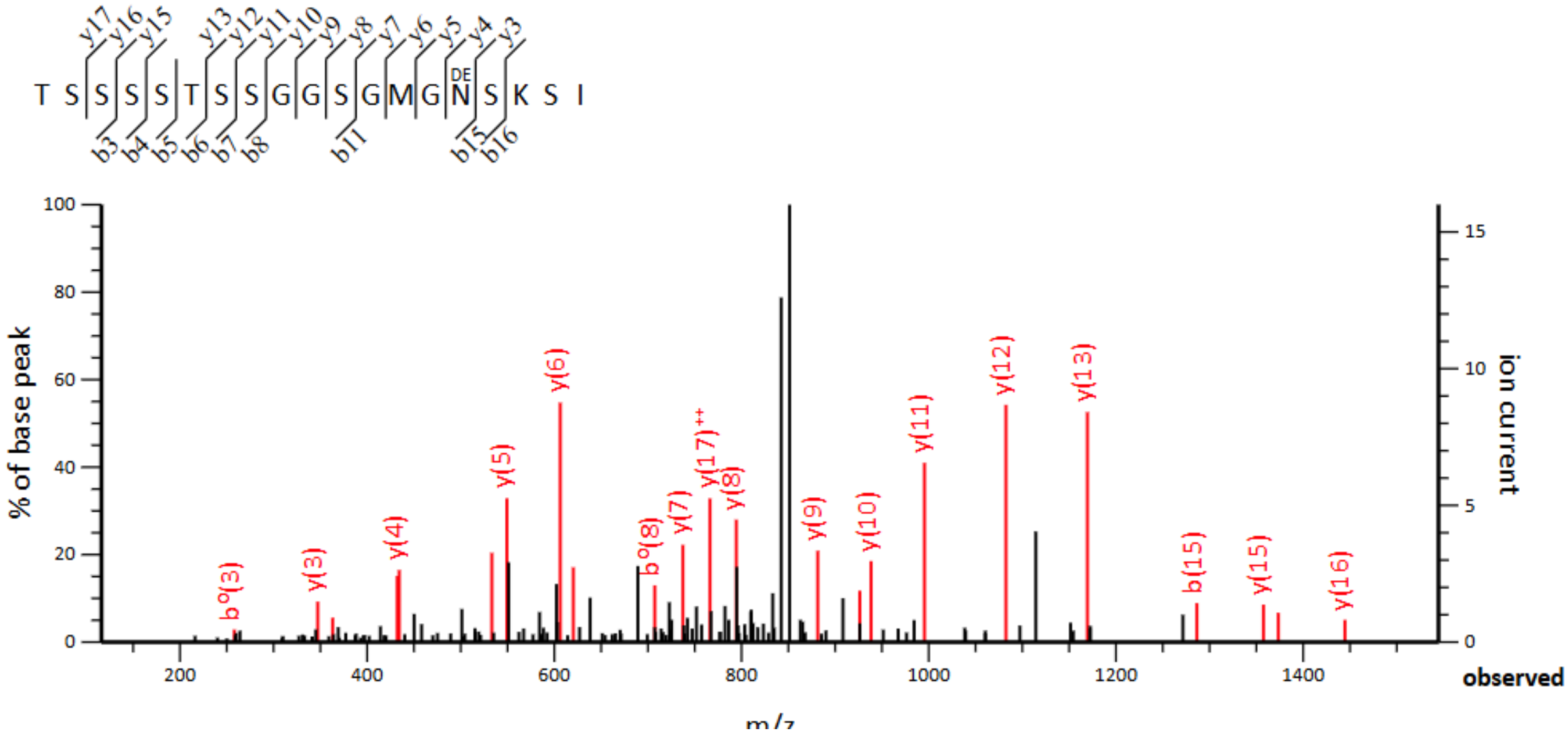

Figure S8. Mass spectrum of the 9FS variant (repeat).

JB6-27FA

7460 69 – 92 685.6428 2053.9067 2053.8937 6.31 0 51 7.7e-06 1 U Y.GKEGLNGGGGGGSHADSPAEAGATA
+ Deamidated (NQ)

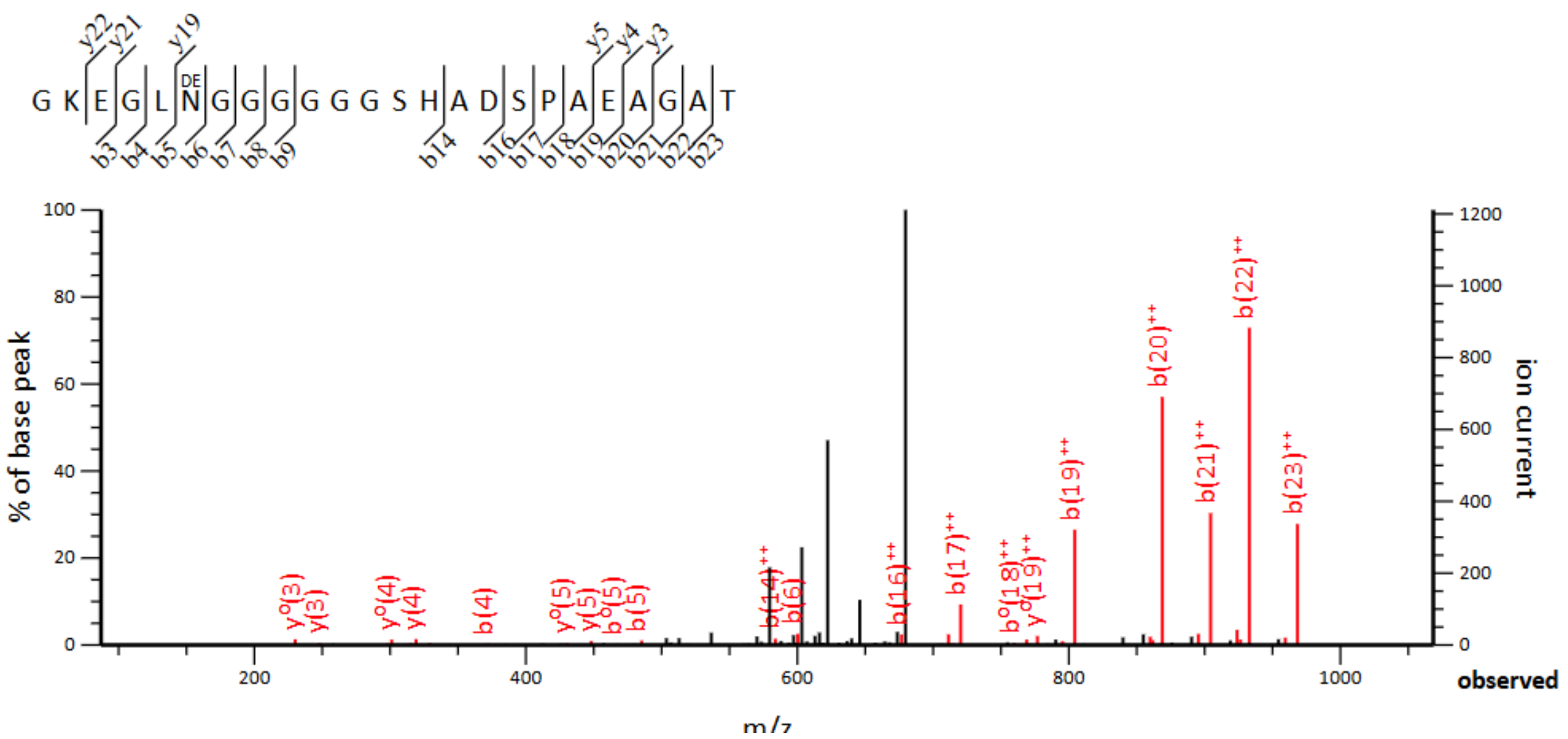

Figure S9. Mass spectrum of the 27FA variant.

2828 93 – 104 428.8823 1283.6250 1283.6218 2.51 0 55 3.5e-06 1 U T.ARNPDDVAREAA.G

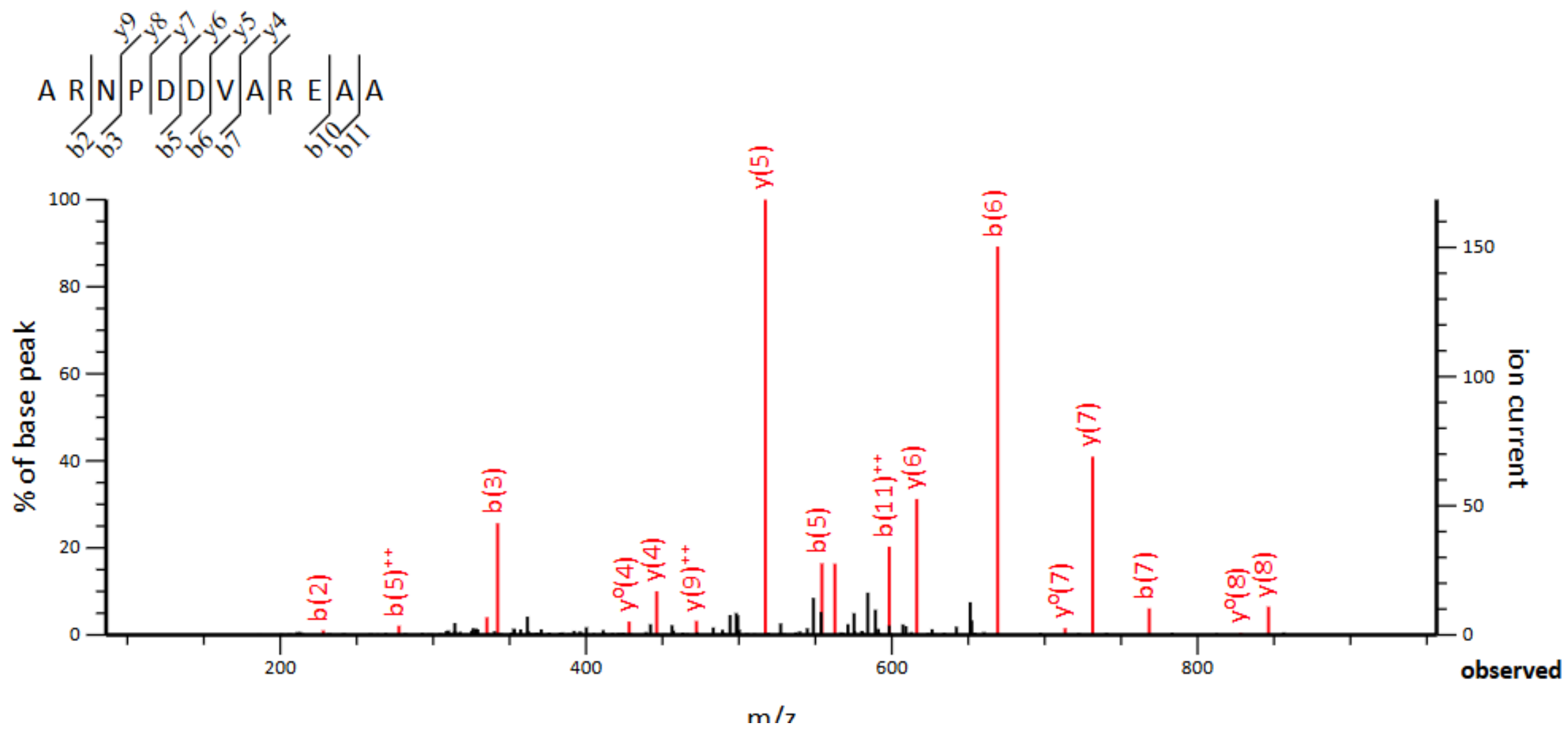

Figure S10. Mass spectrum of the 27FA variant (repeat).

9604 102 – 125 1114.4738 2226.9330 2226.9261 3.13 0 90 9.2e-10 1 U R.EAAGGRDPASADAAEDPAEDAAGN.R

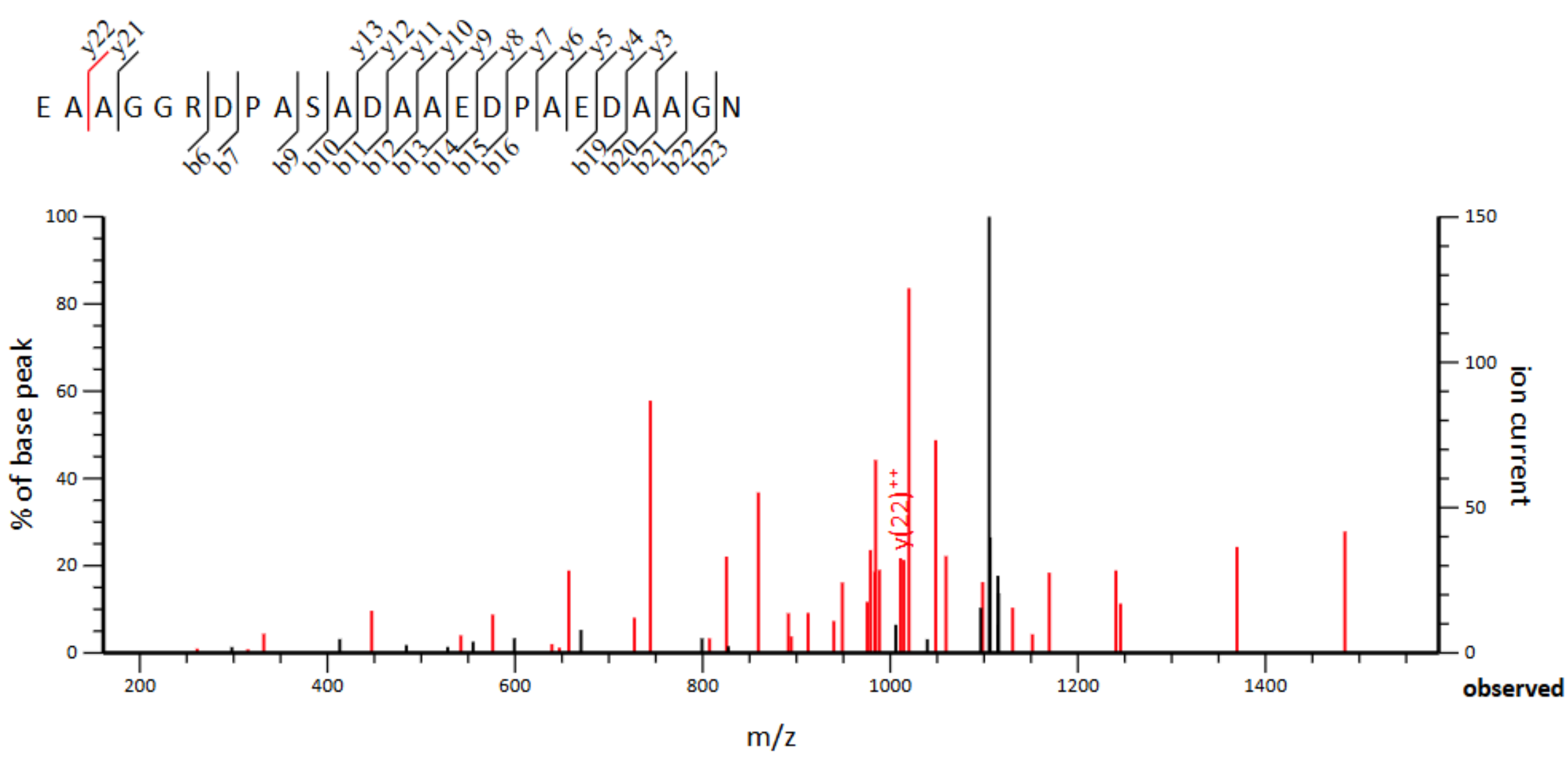

Figure S11. Mass spectrum of the 27FA variant (repeat).

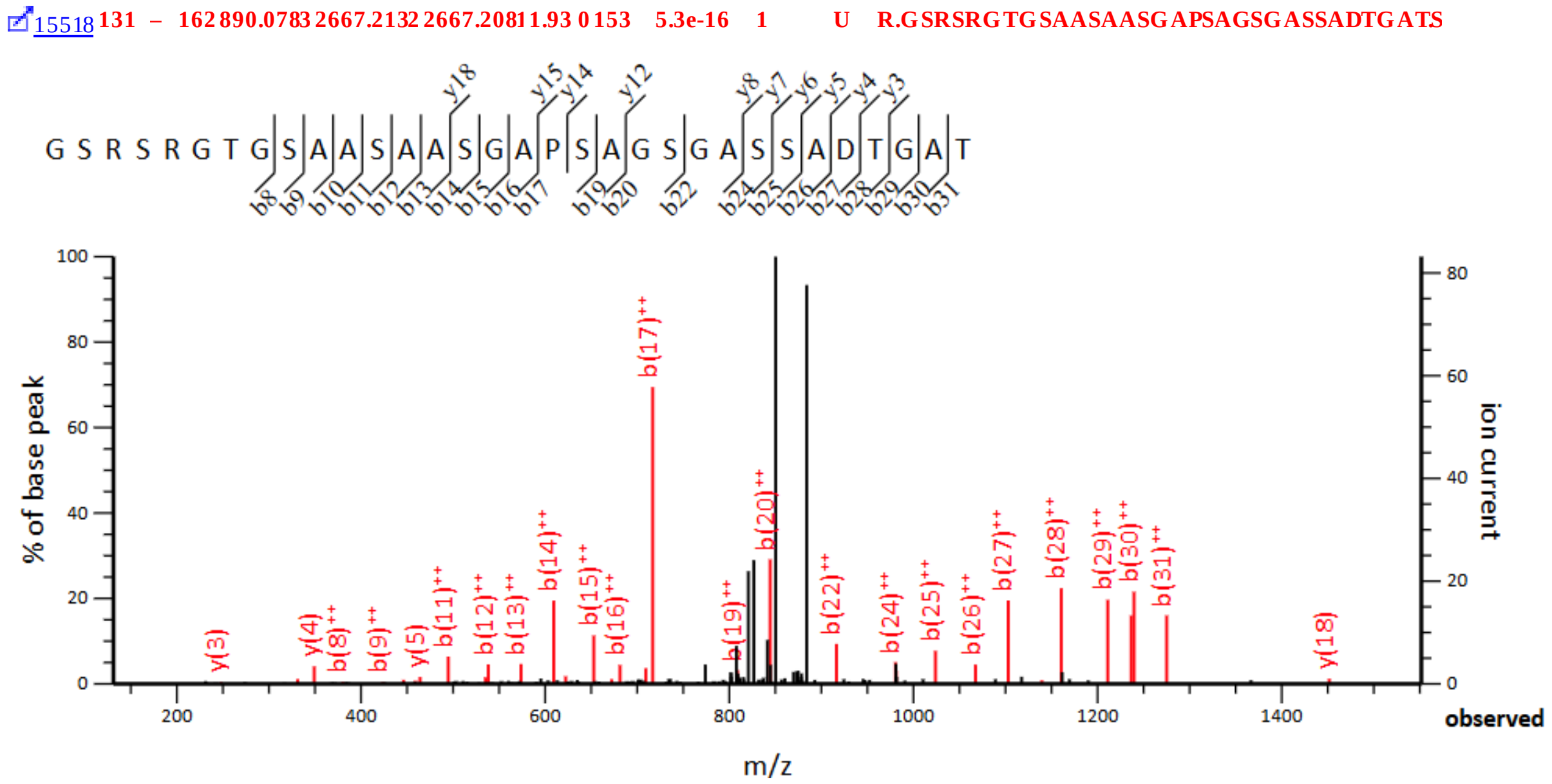

Figure S12. Mass spectrum of the 27FA variant (repeat).

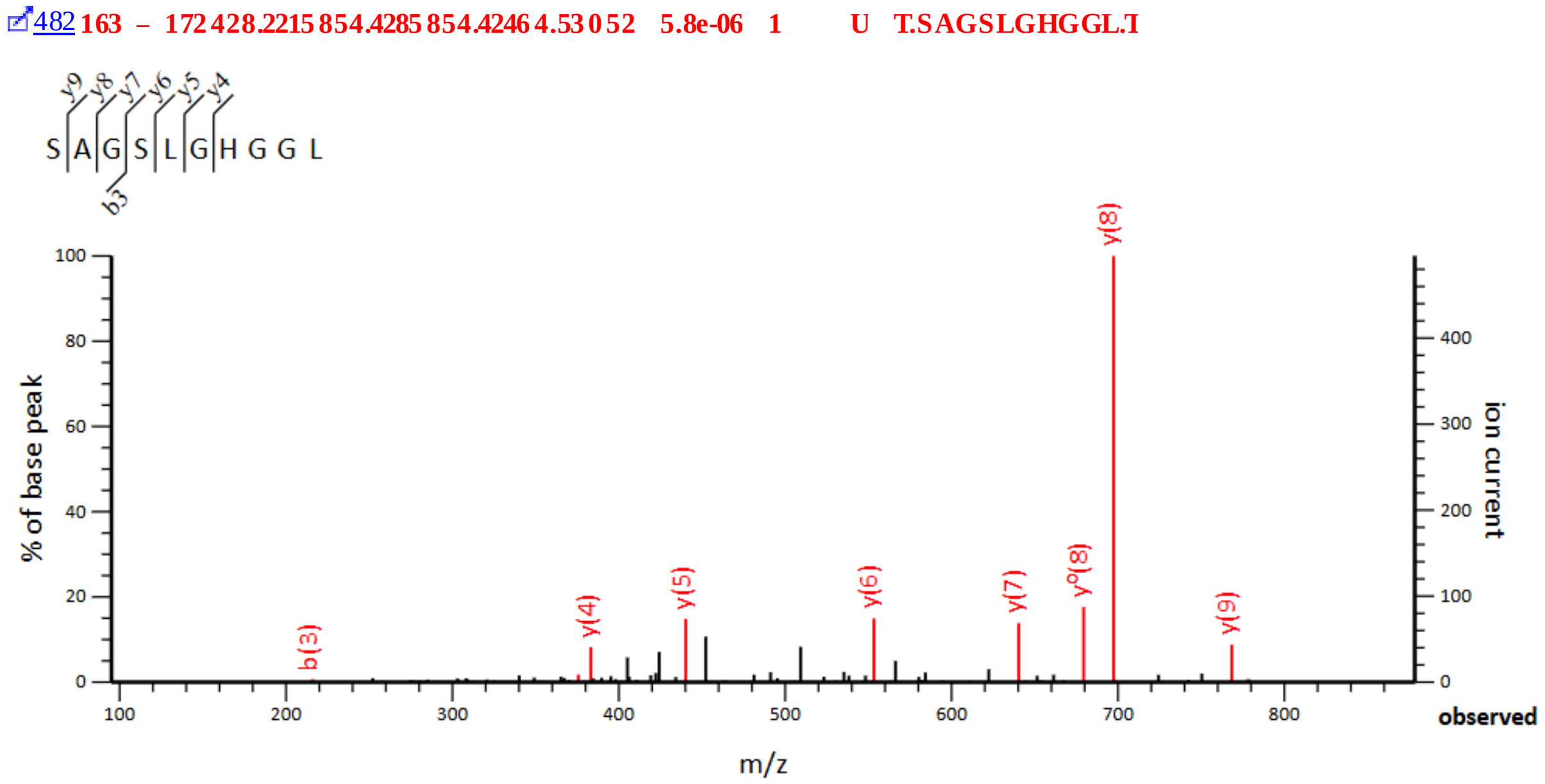

Figure S13. Mass spectrum of the 27FA variant (repeat).

5008 173 – 191 835.8897 1669.7649 1669.7577 4.28 0 91 1.1e-09 1 U L.TSASSTSAGGSGMGNAKSI.S

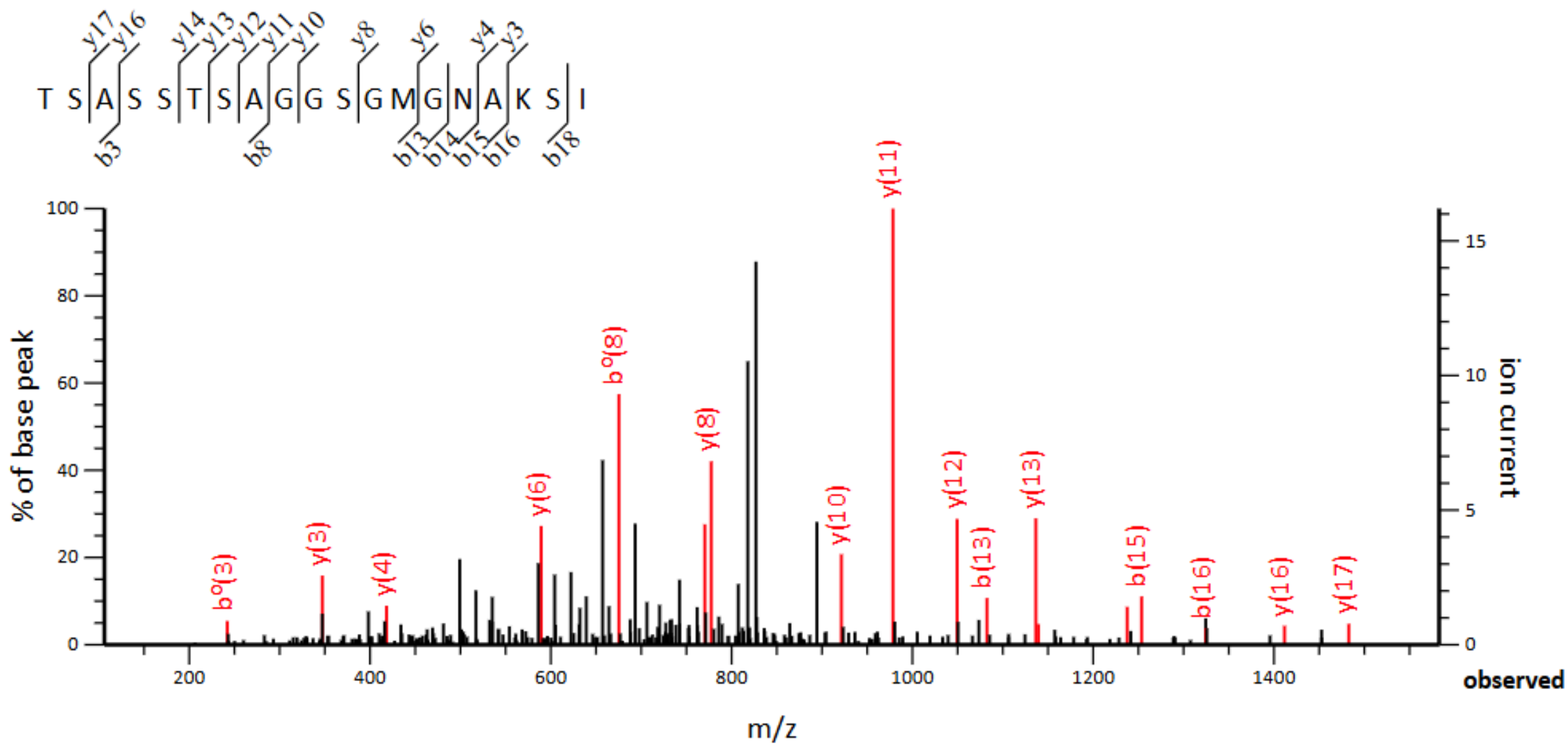

Figure S14. Mass spectrum of the 27FA variant (repeat).

JB6_27FS

15854 69 – 103 673.0904 3360.4154 3360.4323 -5.01 0 42 5.7e-05 1 U Y.GKEGLNGGGGGGSHSDSPSESGSTSRNPDDVSRES.S
+ Deamidated (NQ)

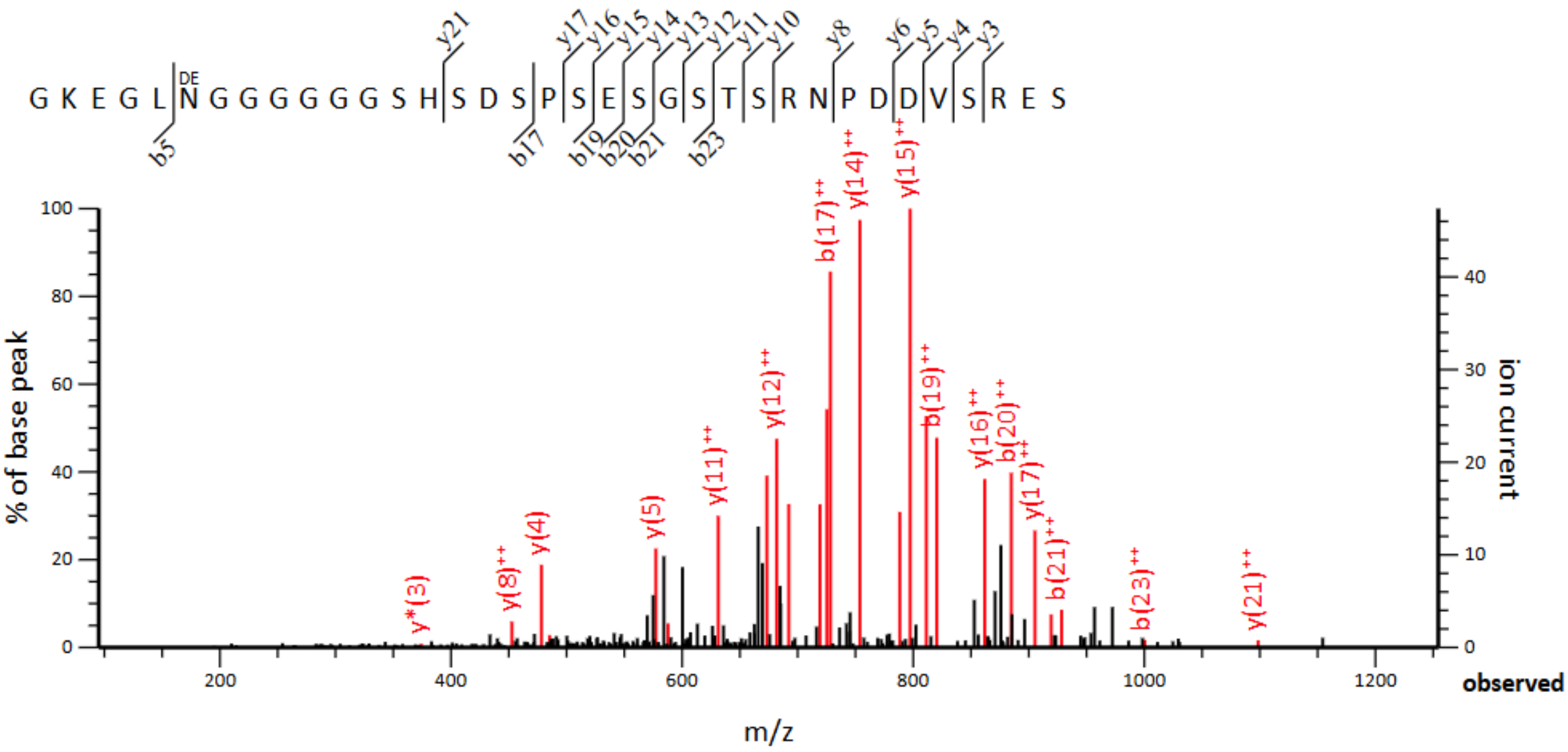

Figure S15. Mass spectrum of the 27FS variant.

8340 104 – 125 1078.4017 2154.7888 2154.8057 -7.84 0 40 0.0001 1 U S.SGGRDPSSSDSSEDPSEDSSGN.R

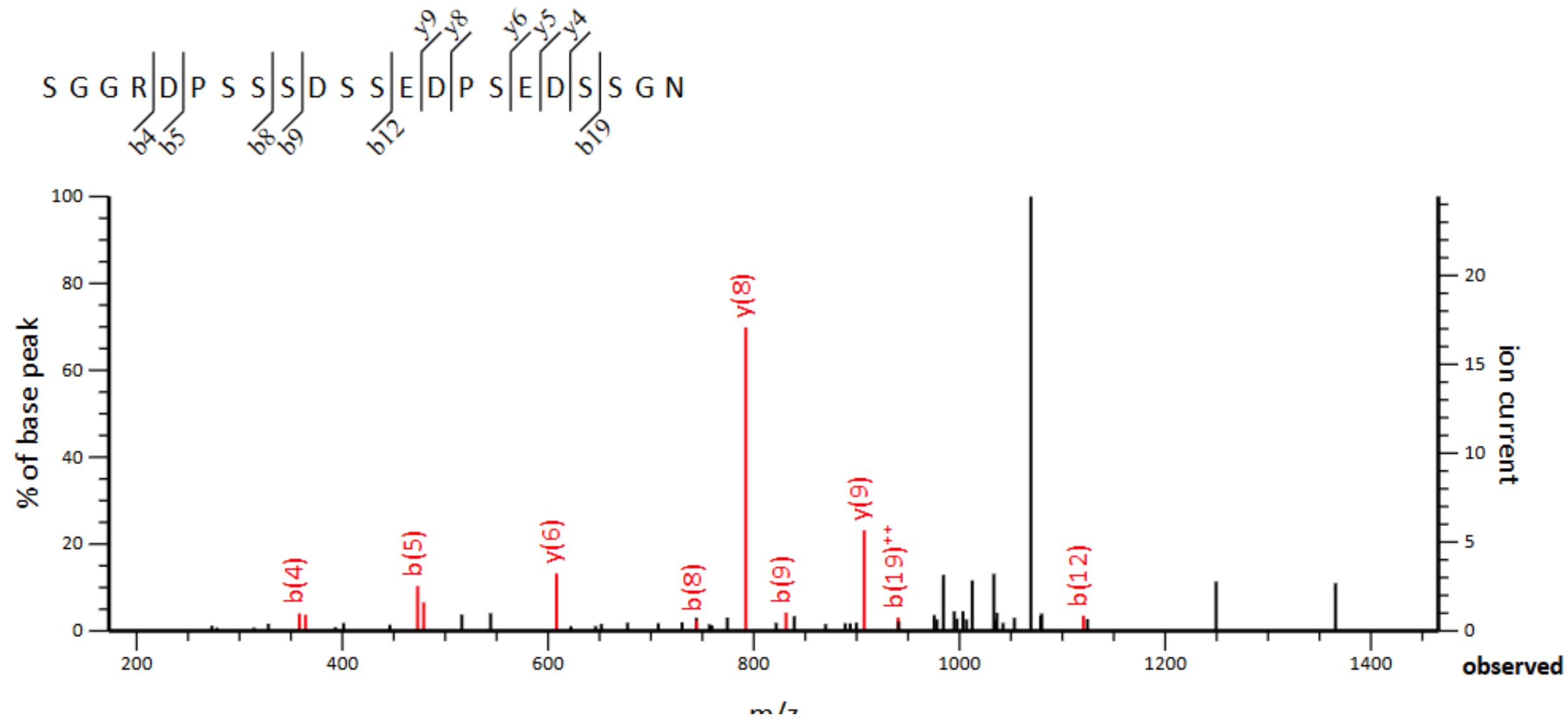

Figure S16. Mass spectrum of the 27FS variant (repeat).

15238 133 – 167 1028.4370 3082.2892 3082.3155 -8.55 0 92 6.3e-10 1 U S.RSRGTGSSSSASSGSPSSGSGSSSSDTGSTSSGSL.G

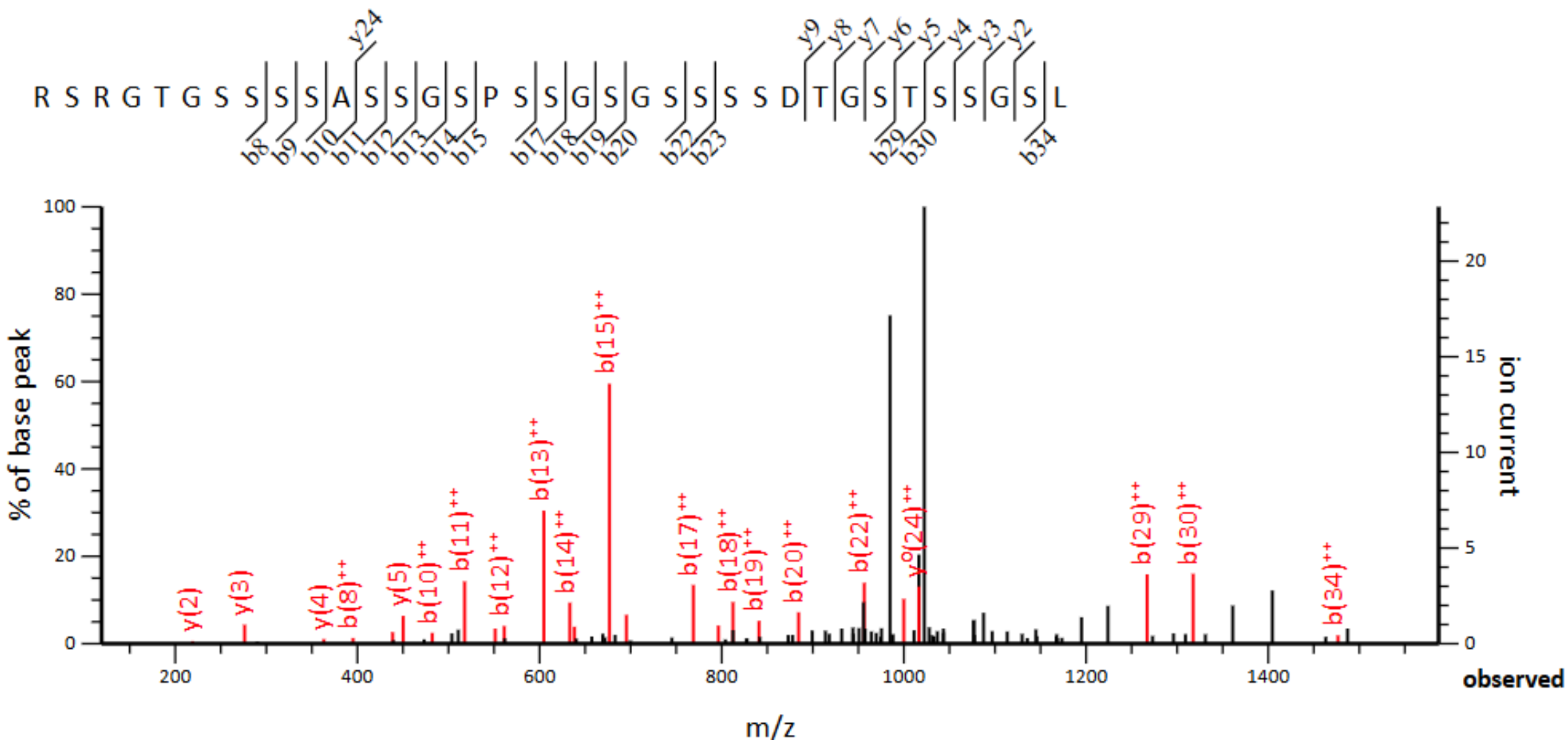

Figure S17. Mass spectrum of the 27FS variant (repeat).

152 164 – 172 392.6987 783.3829 783.3875 -5.90 0 52 6.5e-06 1 S.SGSLGHGGL.T

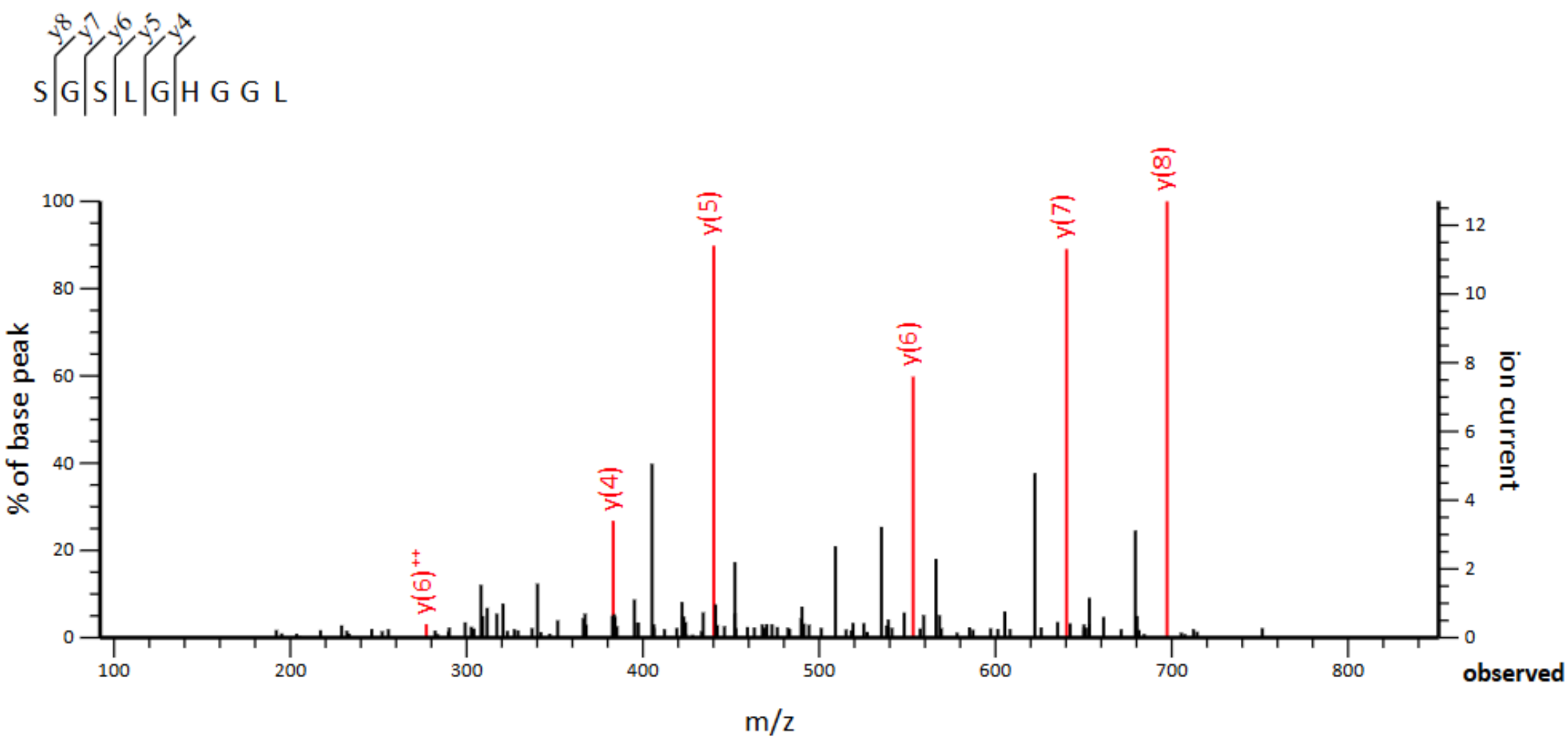

Figure S18. Mass spectrum of the 27FS variant (repeat).

11284 173 – 197 785.6759 2354.0059 2354.0213 -6.55 0 86 2.8e-09 1 U L.TSSSSTSSGGSGMGNSKSISTSTKM.V
+ Deamidated (NQ)

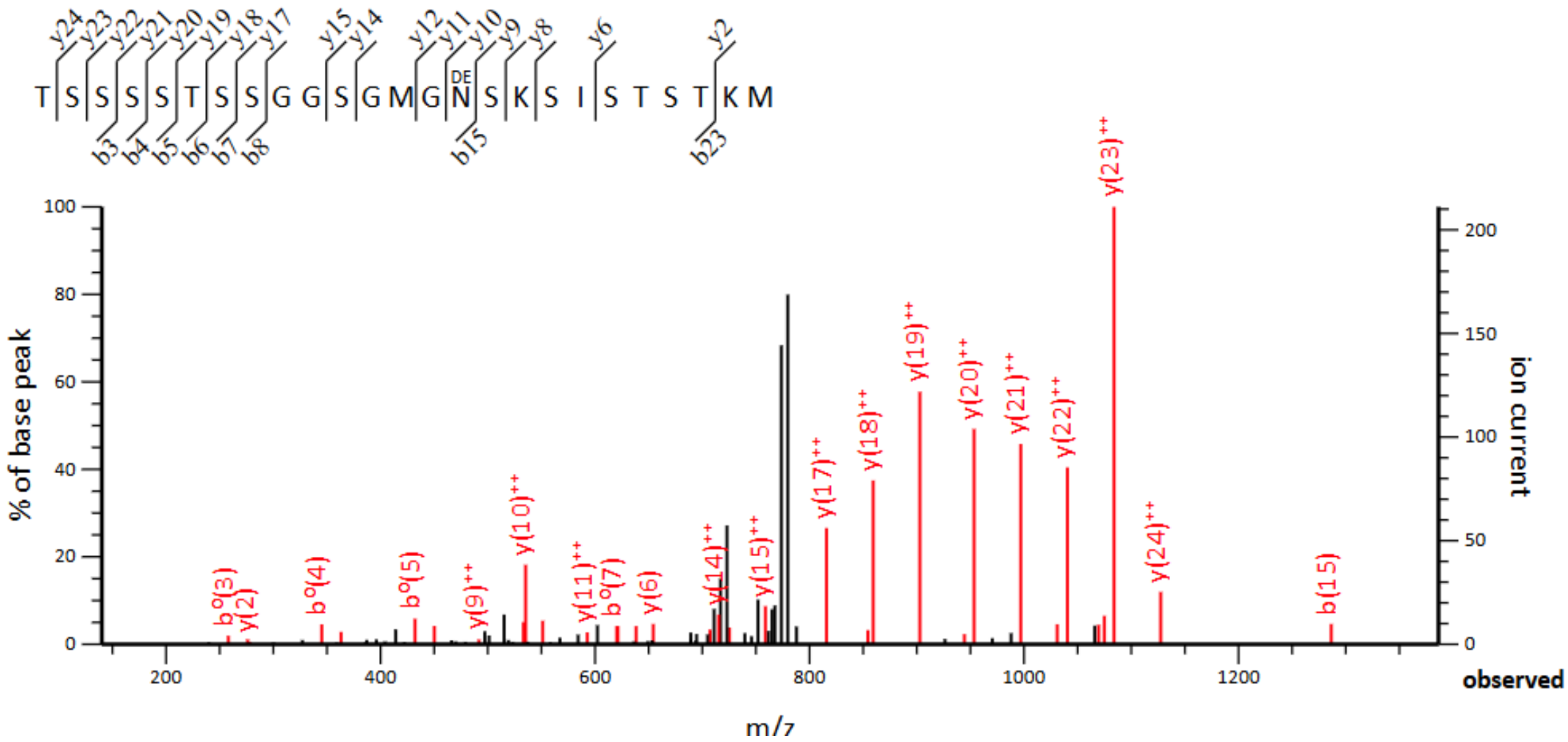

Figure S19. Mass spectrum of the 27FS variant (repeat).

Purification procedure

JB6_3FA

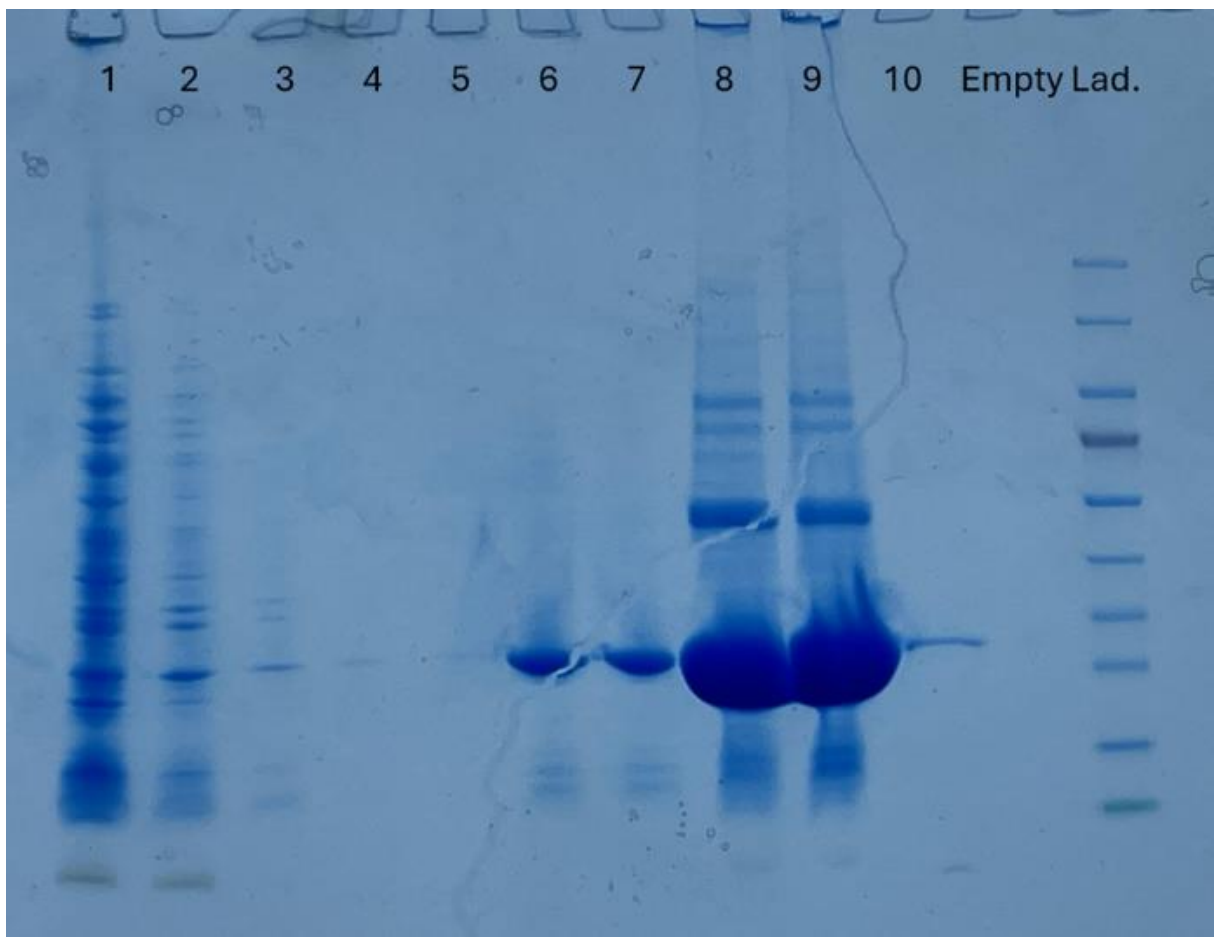

Figure S20. Coomassie-stained 4-20% TRIS/Glycine gel of DNAJB6 variant F3A. Wells 1-5: Supernatants 1-5 from each sonication sequentially. Well 6: FT of Q big beads IEX. Well 7: FT of SPHP IEX. Well 8: Scraped pellet after precipitation with 15% AMS. Well 9: pellet after precipitation with 22% AMS. Well 10: Discarded supernatant after precipitation with 22% AMS.

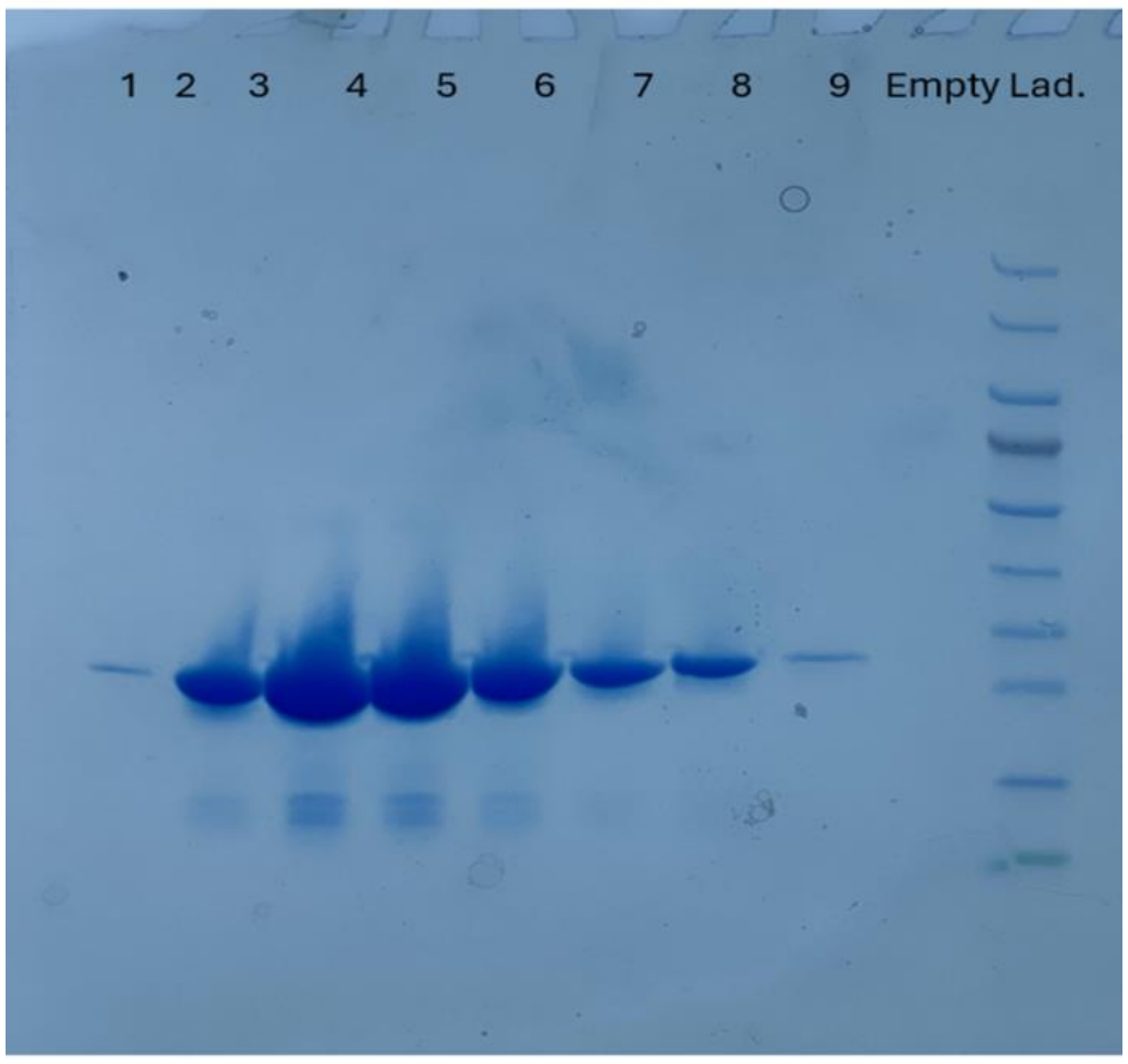

Figure S21. Coomassie-stained 4-20% TRIS/Glycine gel of the first SEC run for the F3A variant. Fractions collected, depicted on gel.

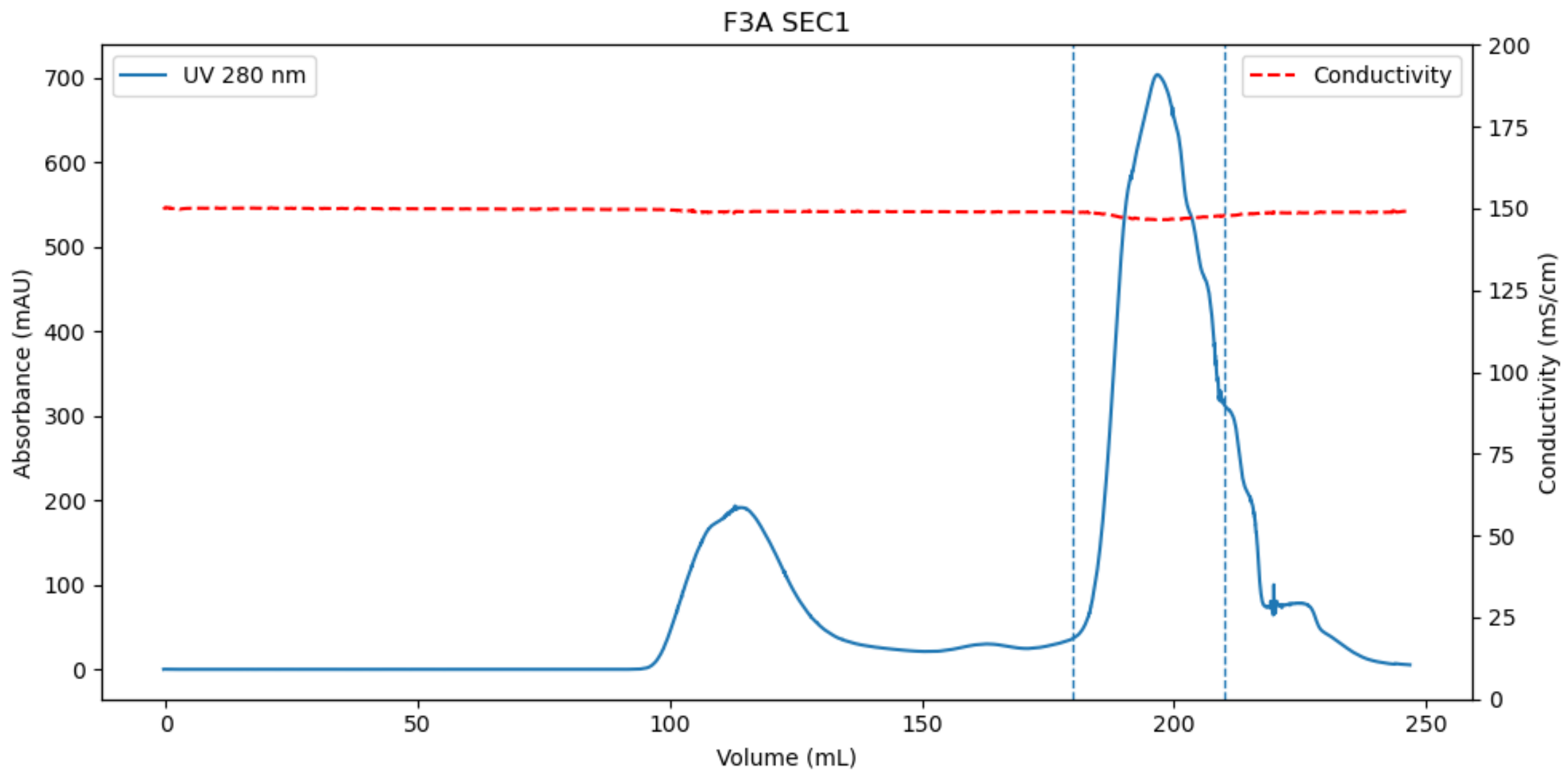

Figure S22. Chromatogram of the first SEC run of the F3A variant. Fractions collected at 180ml-210ml. See supplementary fig. 21 for the gel of collected Fractions.

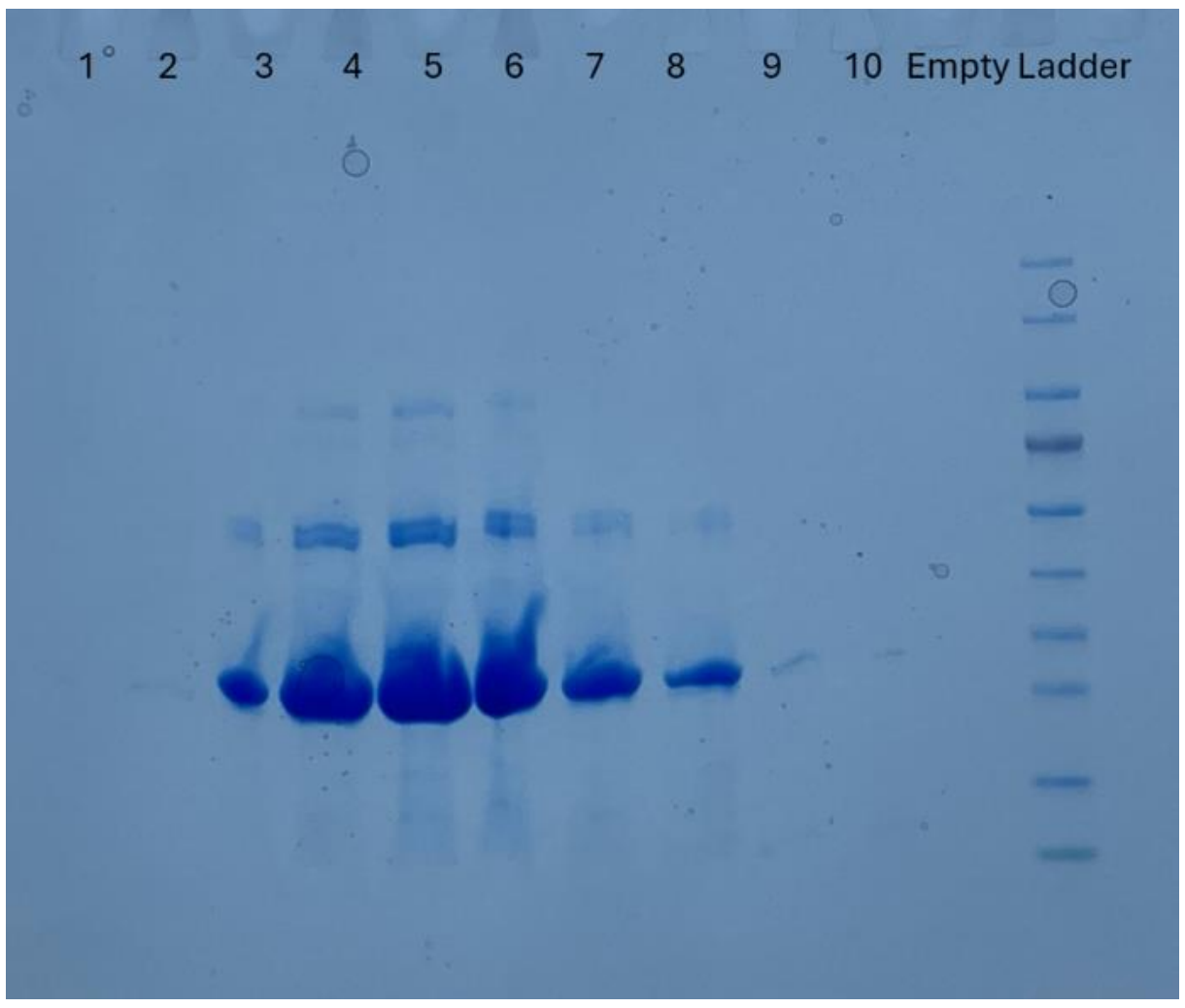

Figure S23. Coomassie-stained 4-20% TRIS/Glycine gel of the second SEC run for the F3A variant. Fractions collected, depicted on gel (3-7).

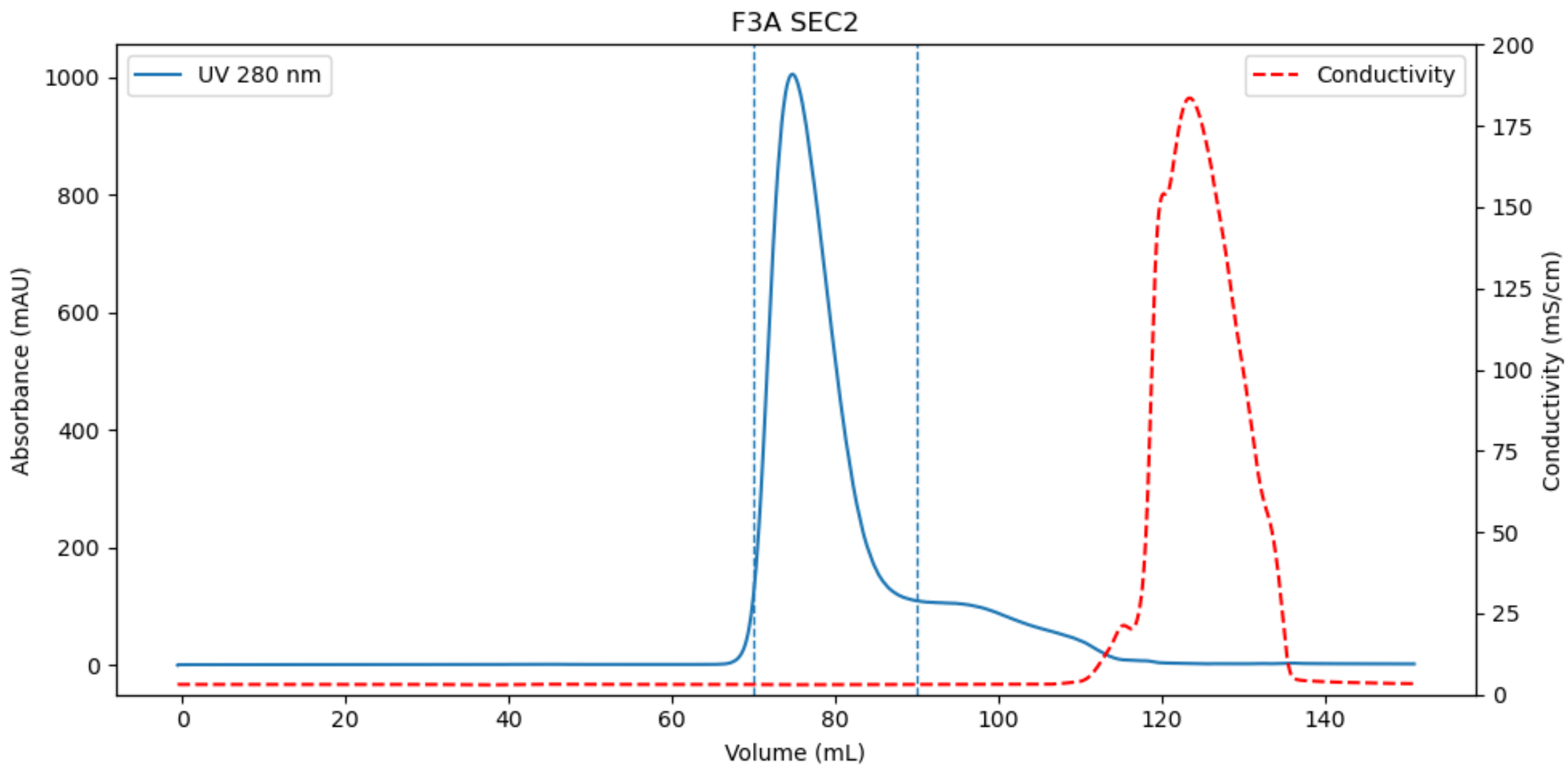

Figure S24. Chromatogram of the second SEC run for F3A. Fractions from 70-90 ml were collected. See supplementary fig. 23 for the gel of collected Fractions.

JB6_F6A

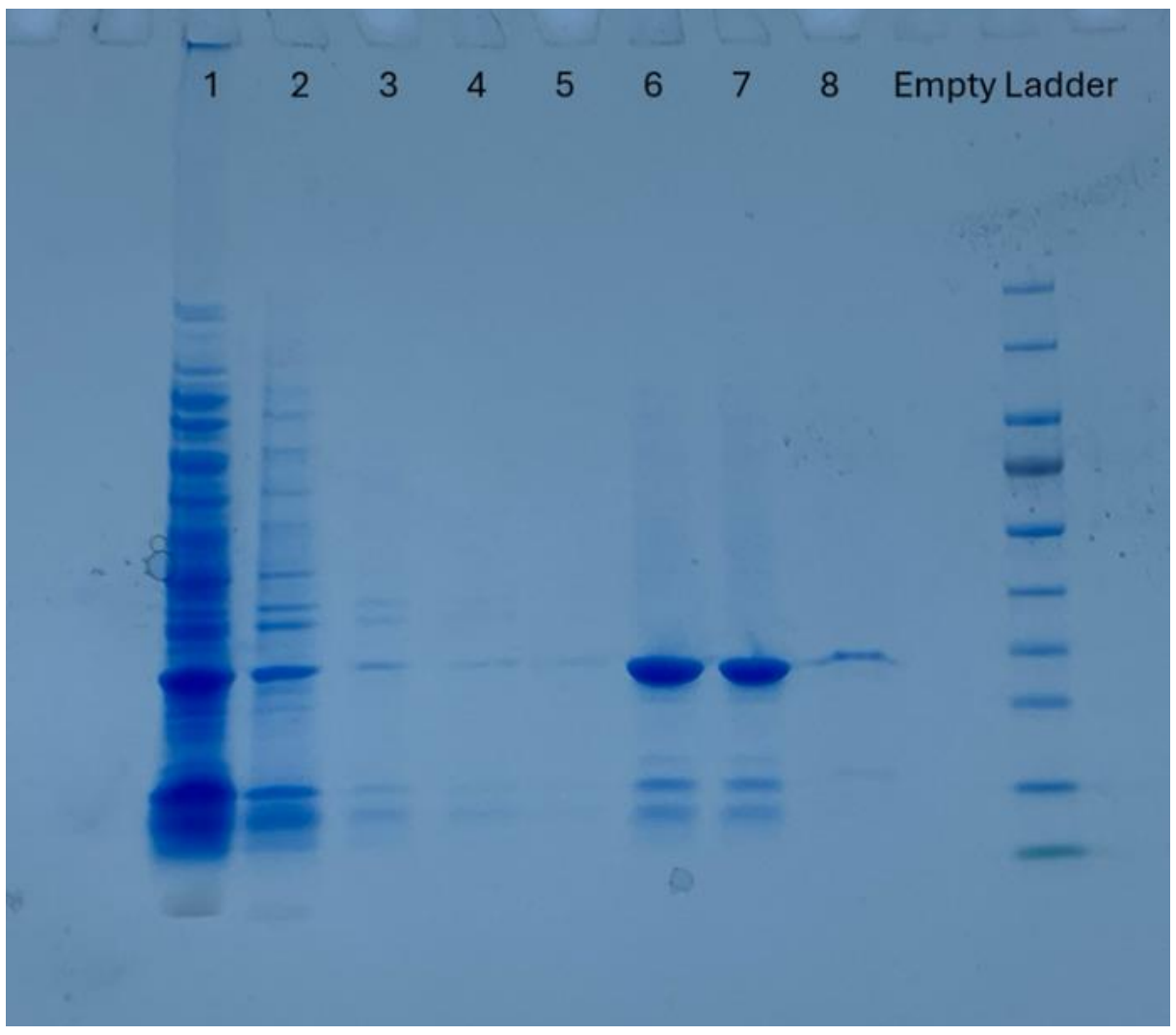

Figure S25. Coomassie-stained 4-20% TRIS/Glycine gel of DNAJB6 variant F6A. Wells 1-5: Supernatants 1-5 from each sonication sequentially. Well 6: FT of Q big beads IEX. Well 7: FT of SPHP IEX. Well 8: Scraped pellet after precipitation with 22% AMS

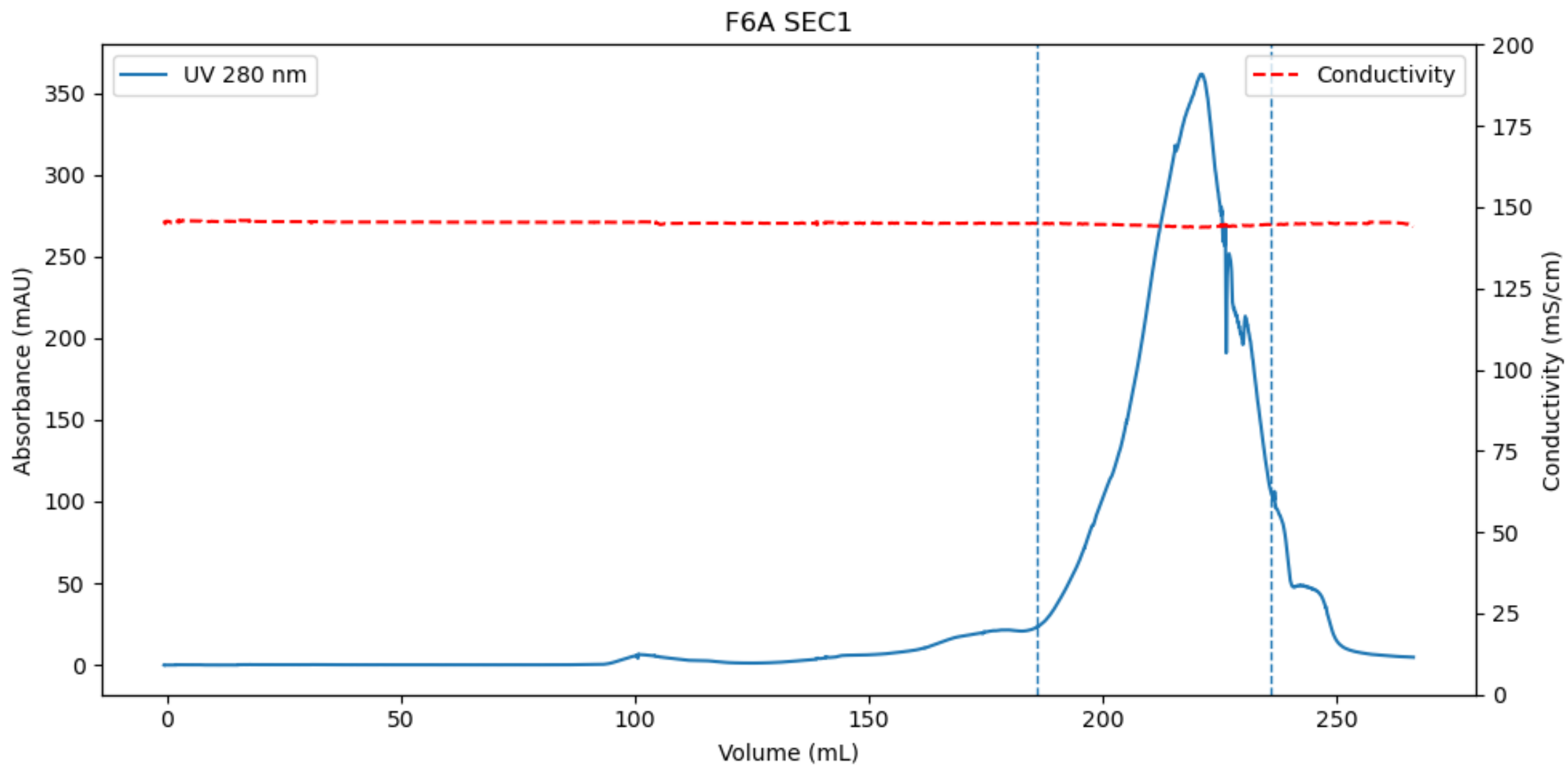

Figure S26. Chromatogram of the first SEC run of the F6A variant. Fractions were collected at 186-236 ml. All fractions were used for the next step.

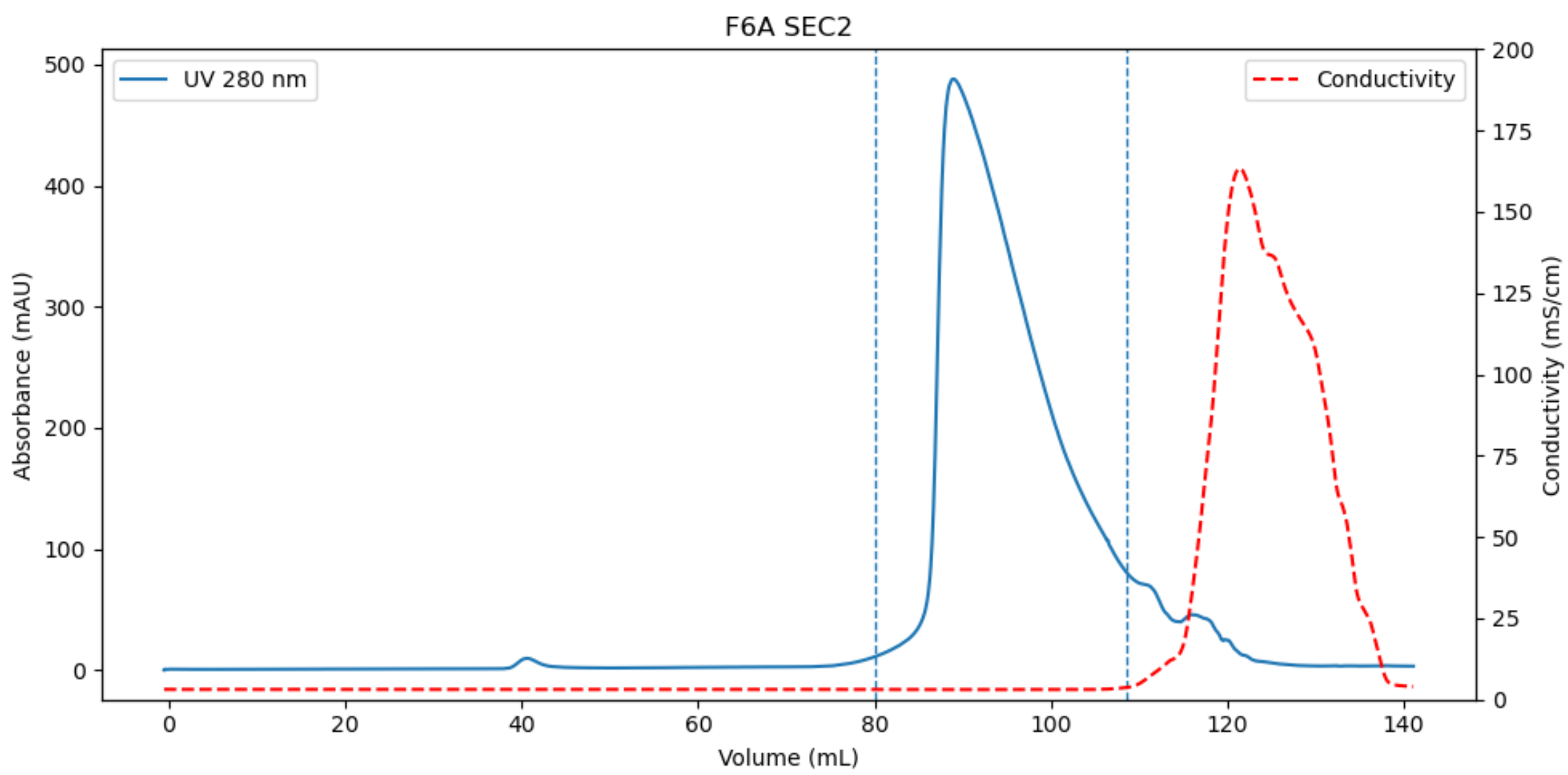

Figure S27. Chromatogram of the second SEC run for F6A. Fractions from 80-108.5 ml were collected. See supplementary fig. 28 for the gel of collected Fractions.

.

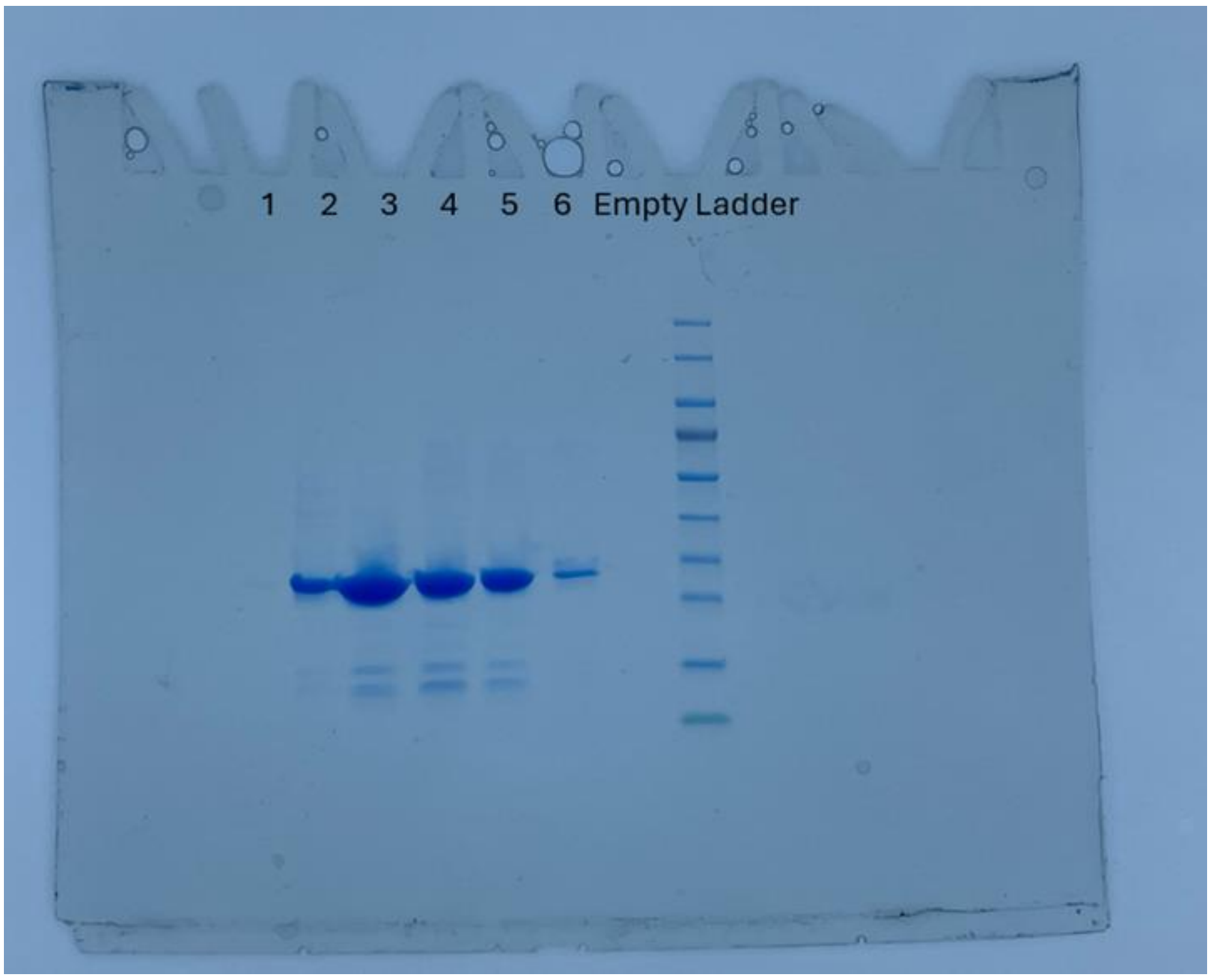

Figure S28. Coomassie-stained 4-20% TRIS/Glycine gel of the second SEC run for the F6A variant. Fractions collected, depicted on gel. Fractions in wells 2-5 were pooled. Due to very high loading concentrations, the degradation bands appear more visible.

Other variants

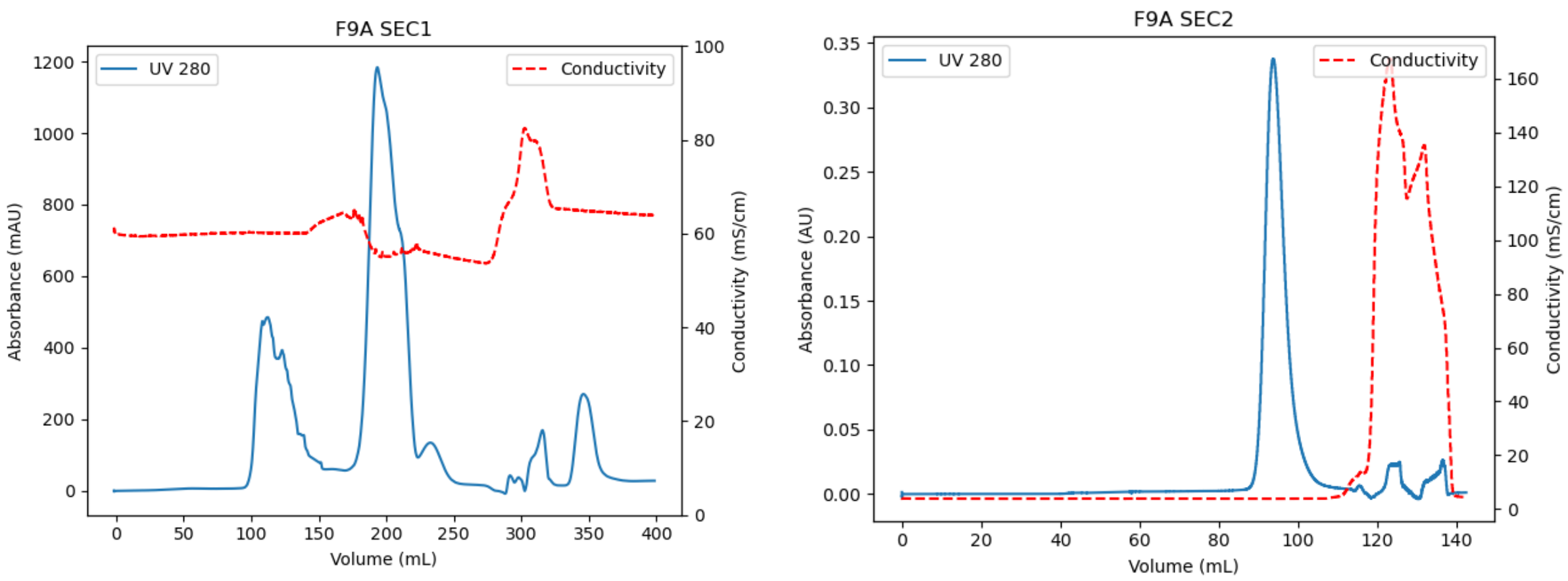

Figure S29. The two SEC run chromatograms for the F9A variant of JB6

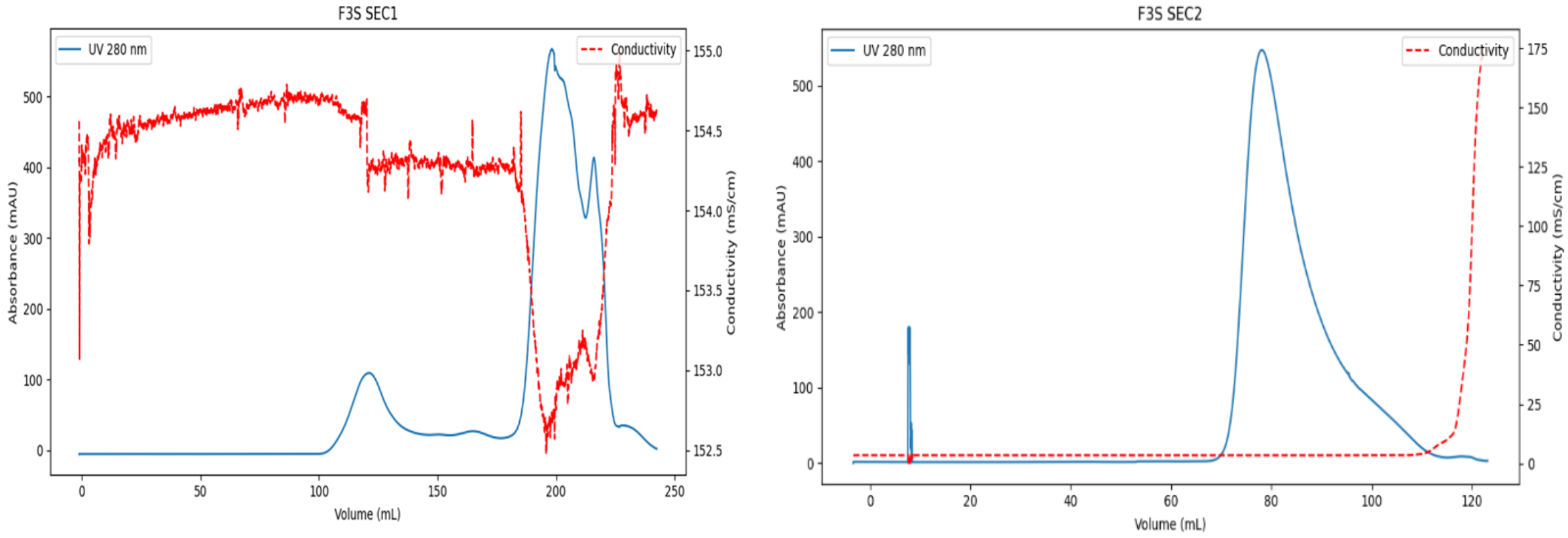

Figure S30. The two SEC run chromatograms for the F3S variant of JB6

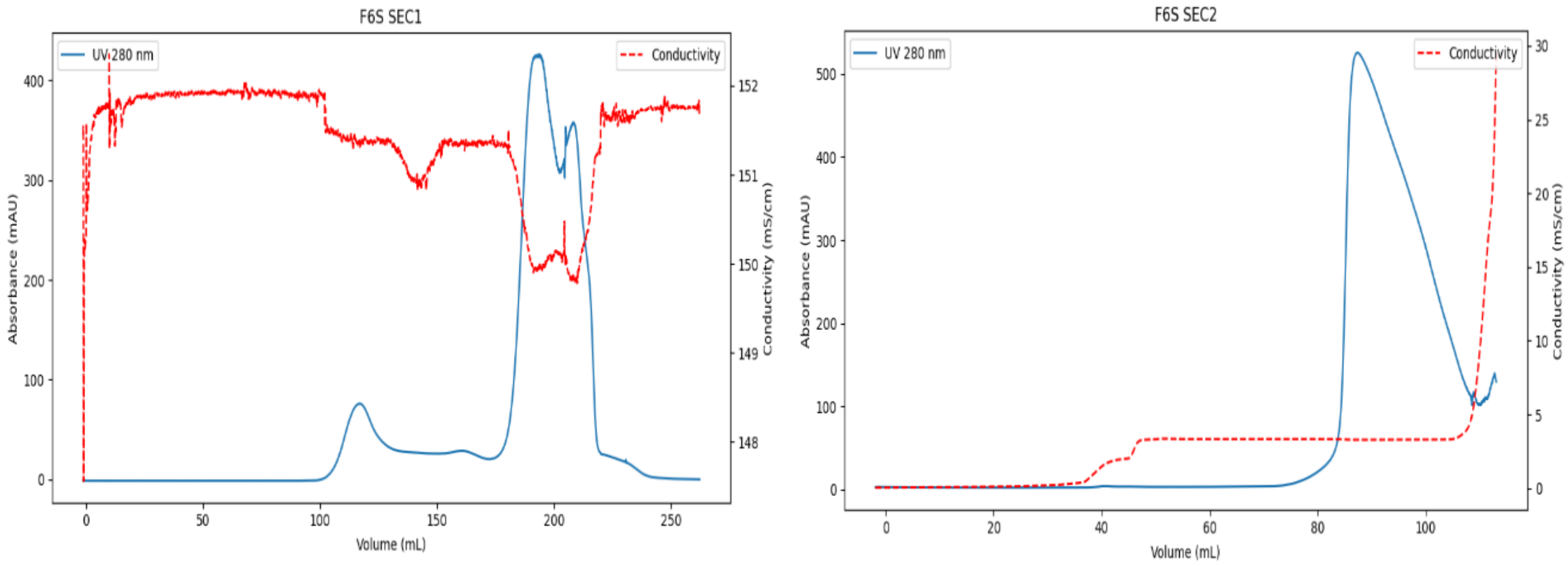

Figure S31. The two SEC run chromatograms for the F6S variant of JB6

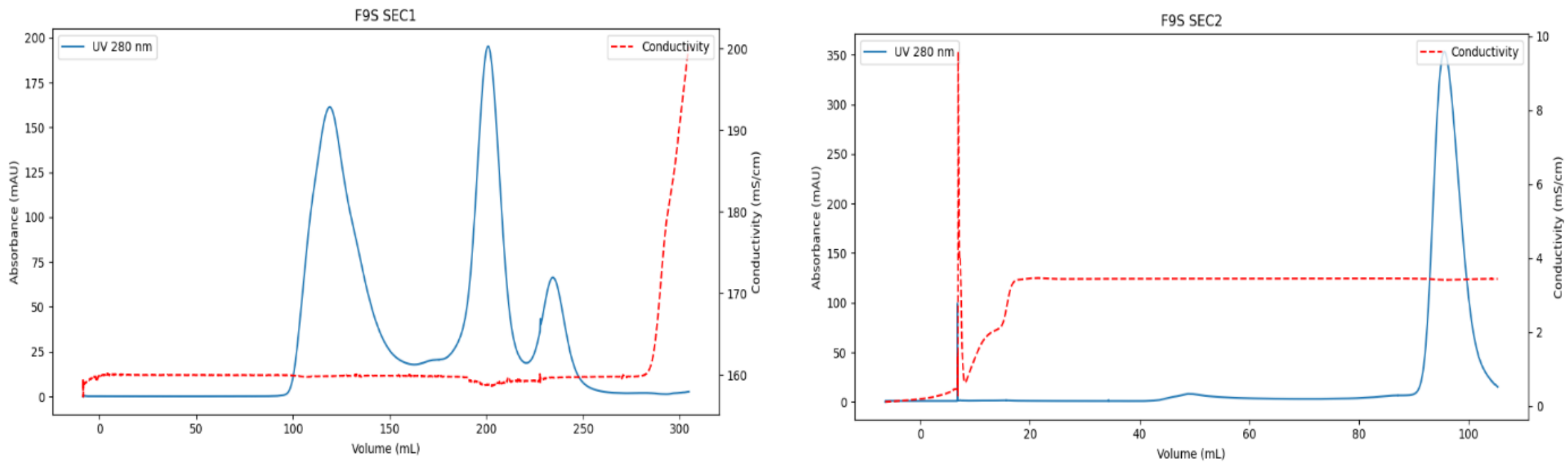

Figure S32. The two SEC run chromatograms for the F9S variant of JB6

Thioflavin T kinetics

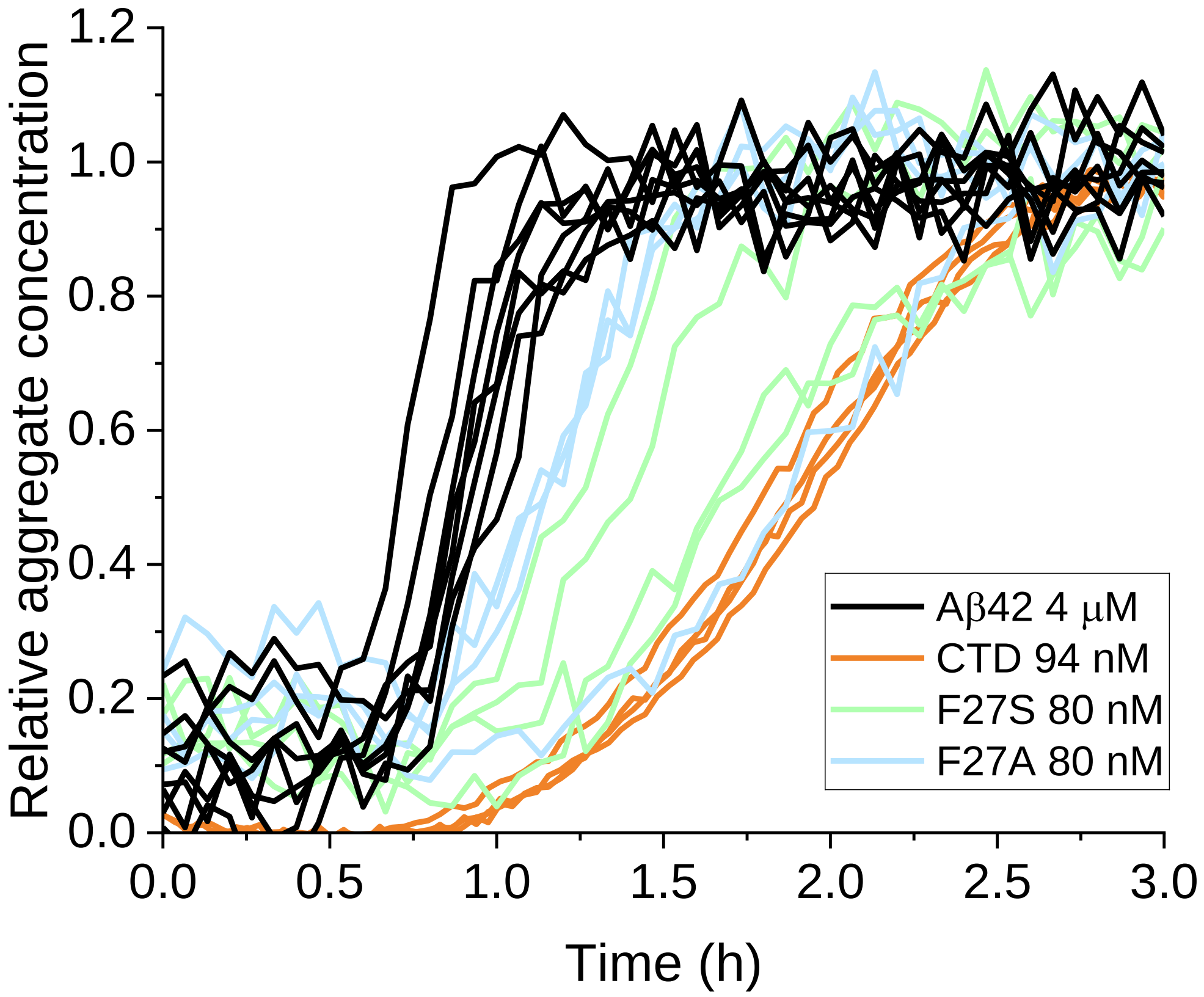

Figure S33. Comparison between Aβ42 aggregation delay by the CTD of DNAJB6 at 94 nM and the variants F27S and F27A at 80 nM. Data for the CTD was taken from Österlund et al. (2023)[1].

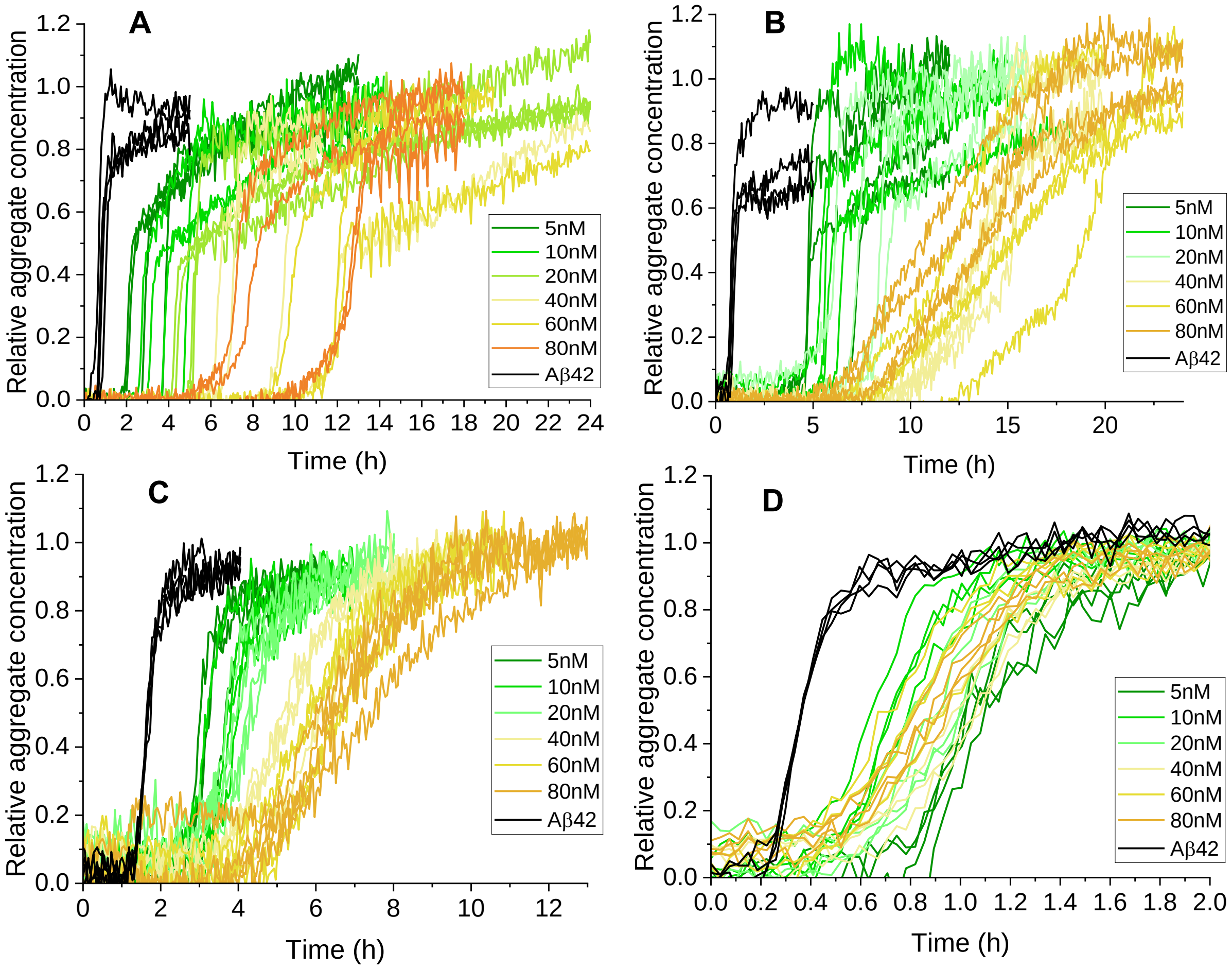

Figure S34. Example thioflavin T fluorescence curves for each F to S substituted variant interacting with Aβ42. A-F3S; B-F6S; C-F9S; D-F27S. The concentration of Aβ42 is 4 μM.

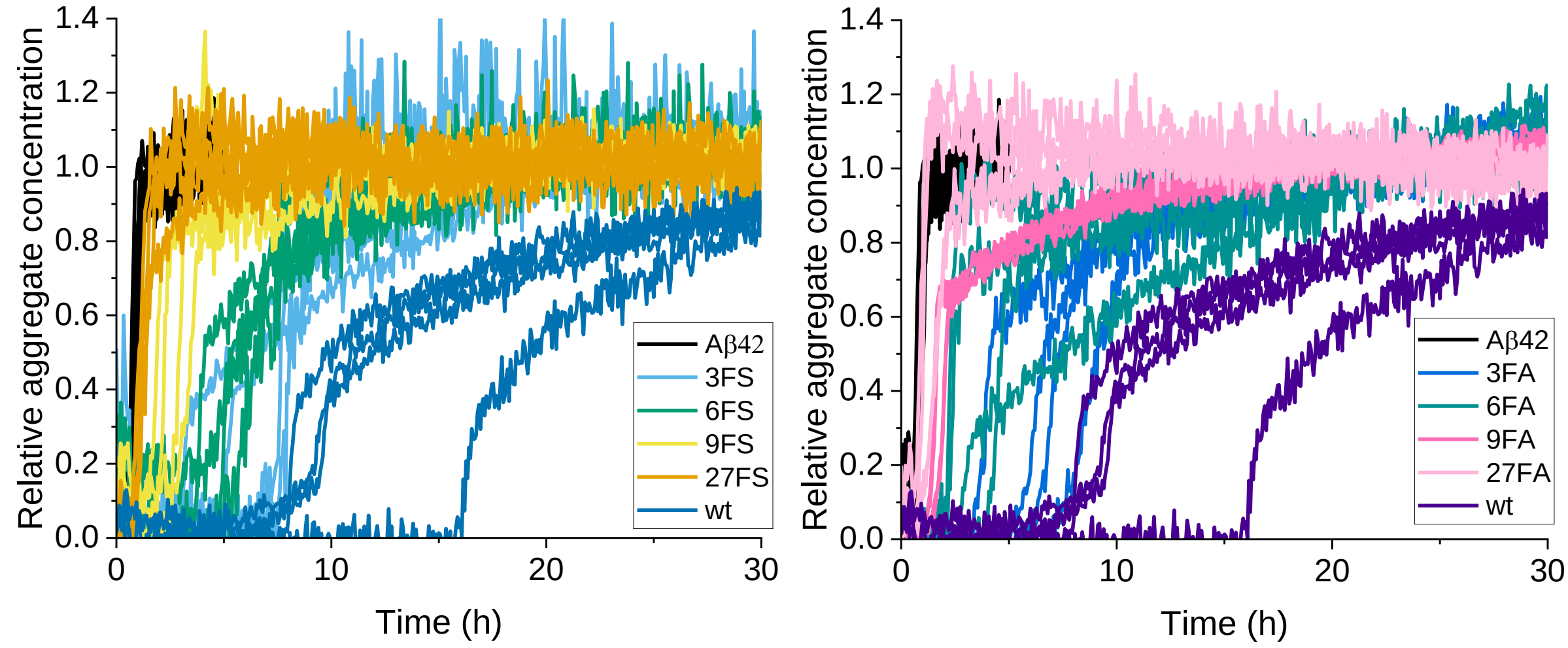

Figure S35. ThT fluorescence versus time for all variants within the same plate at 20 nM. A- F to S variants. B- F to A variants. The concentration of Aβ42 is 4 μM.

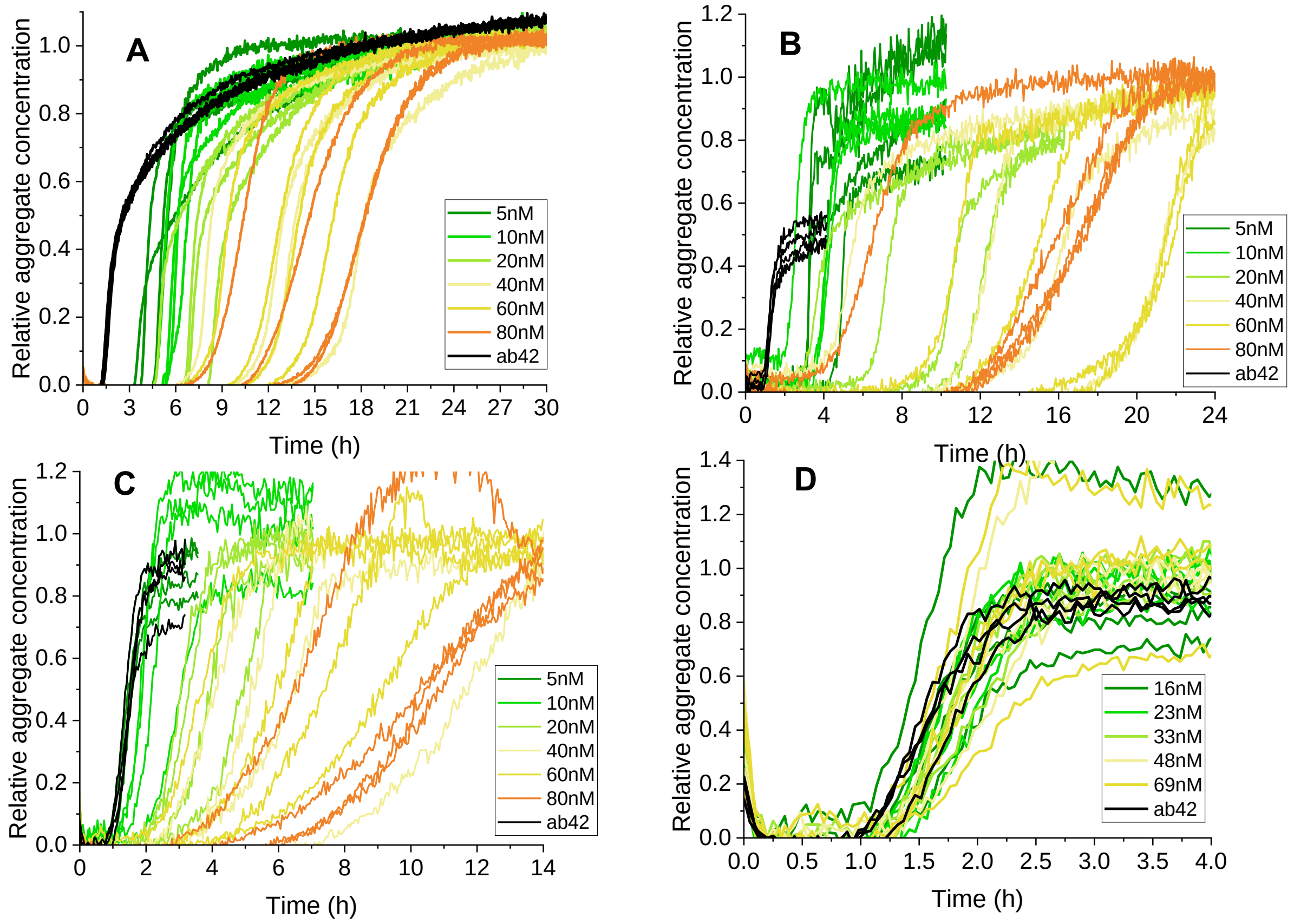

Figure S36. Example thioflavin T fluorescence curves for each F to S substituted variant interacting with Aβ42. A-F3A; B-F6A; C-F9A; D-F27A. The concentration of Aβ42 is 3 μM.

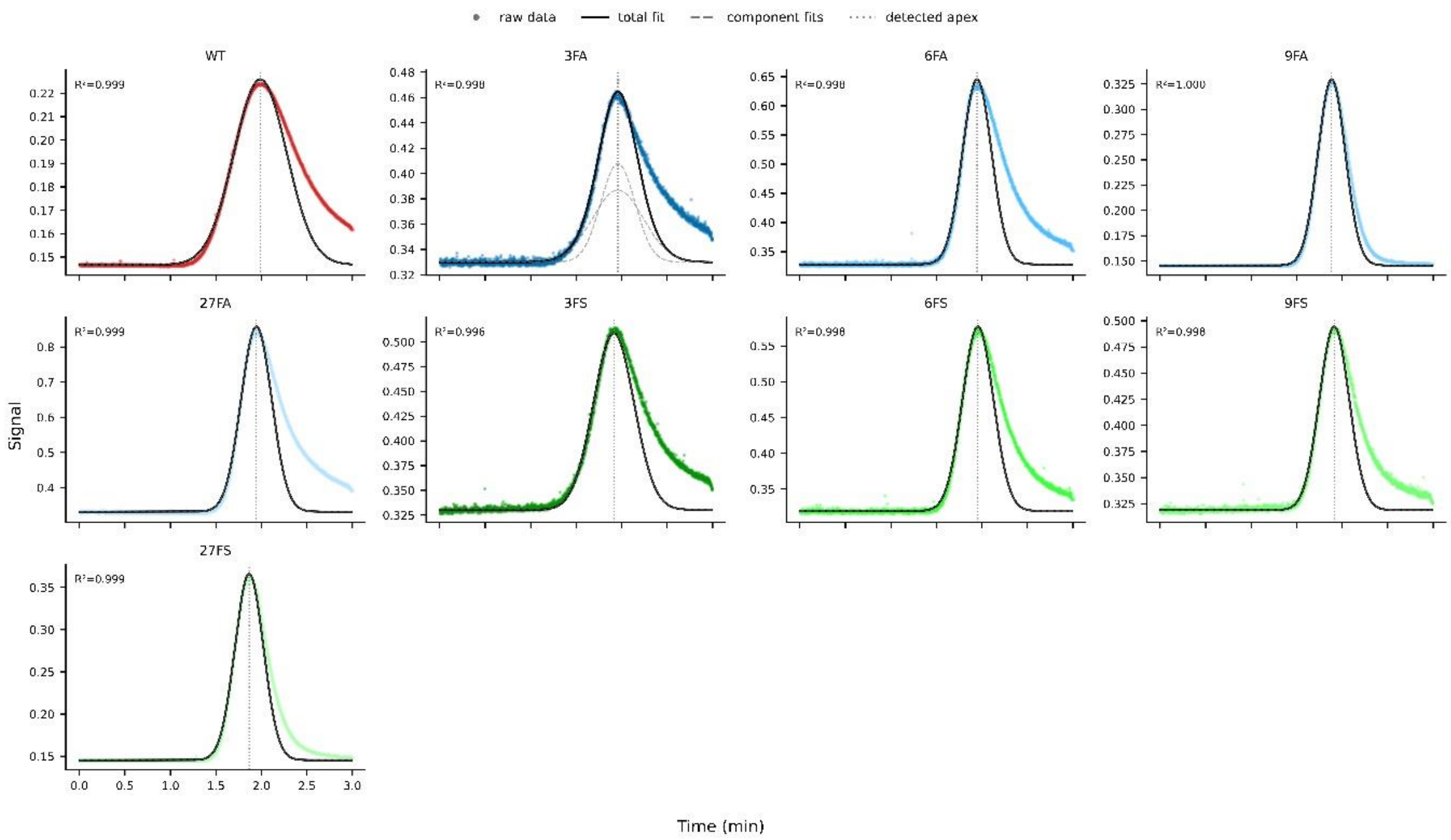

Figure S37. Example FIDA fit for each sample.

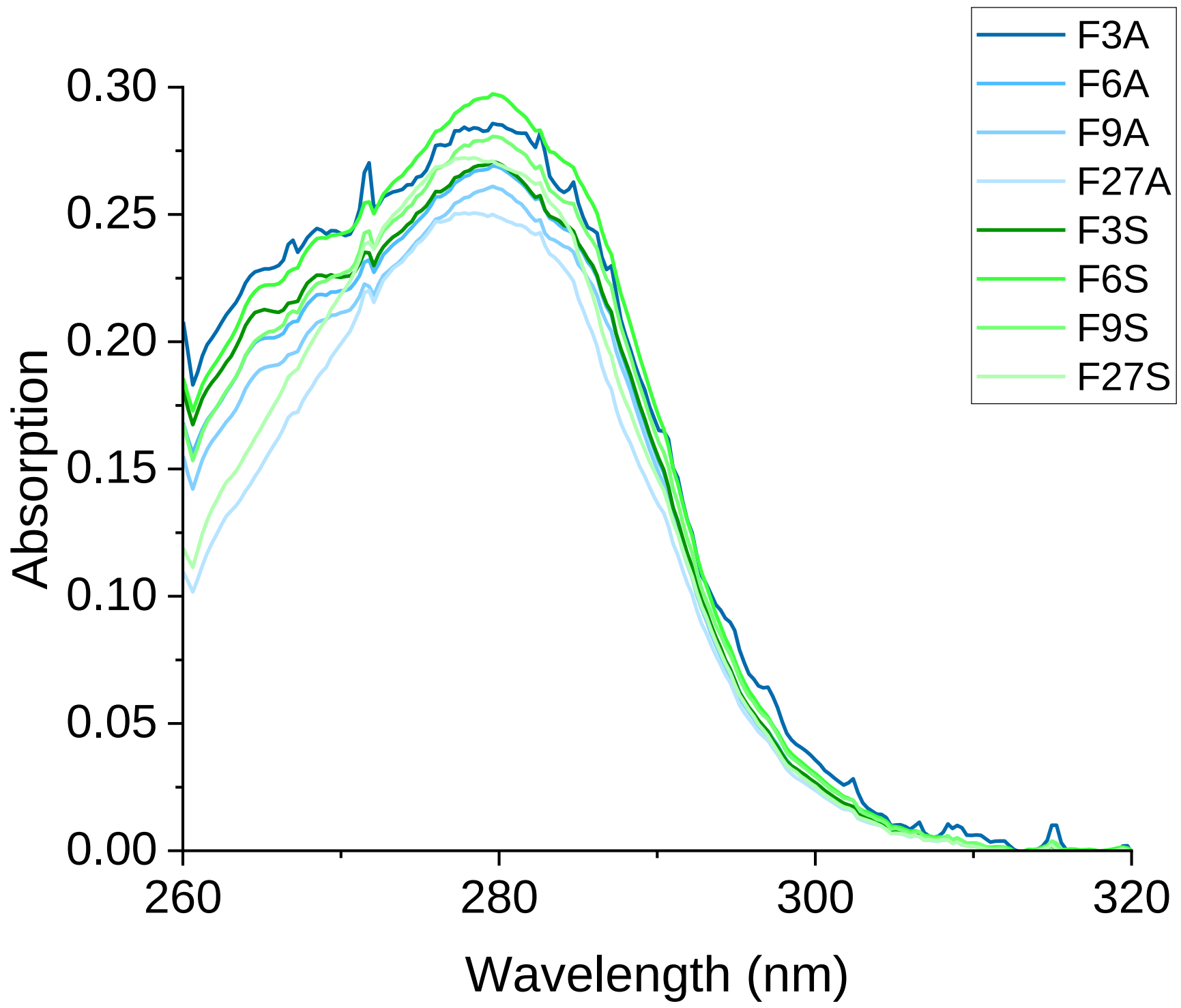

Figure S38. Absorption spectra of each variant measured using a Labbot instrument, with a path length of 1 cm. The samples were prepared at an apparent concentration of 20 µM, based on absorbance during purification, with 2M GuHCl in each sample, the concentrations were then noted based on the absorbance at 280 nm using an extension coefficient of 14400 cm^{-1} M^{-1}. The absorbance from F residues in the 260-270 nm range can be seen to disappear with increasing substitutions.

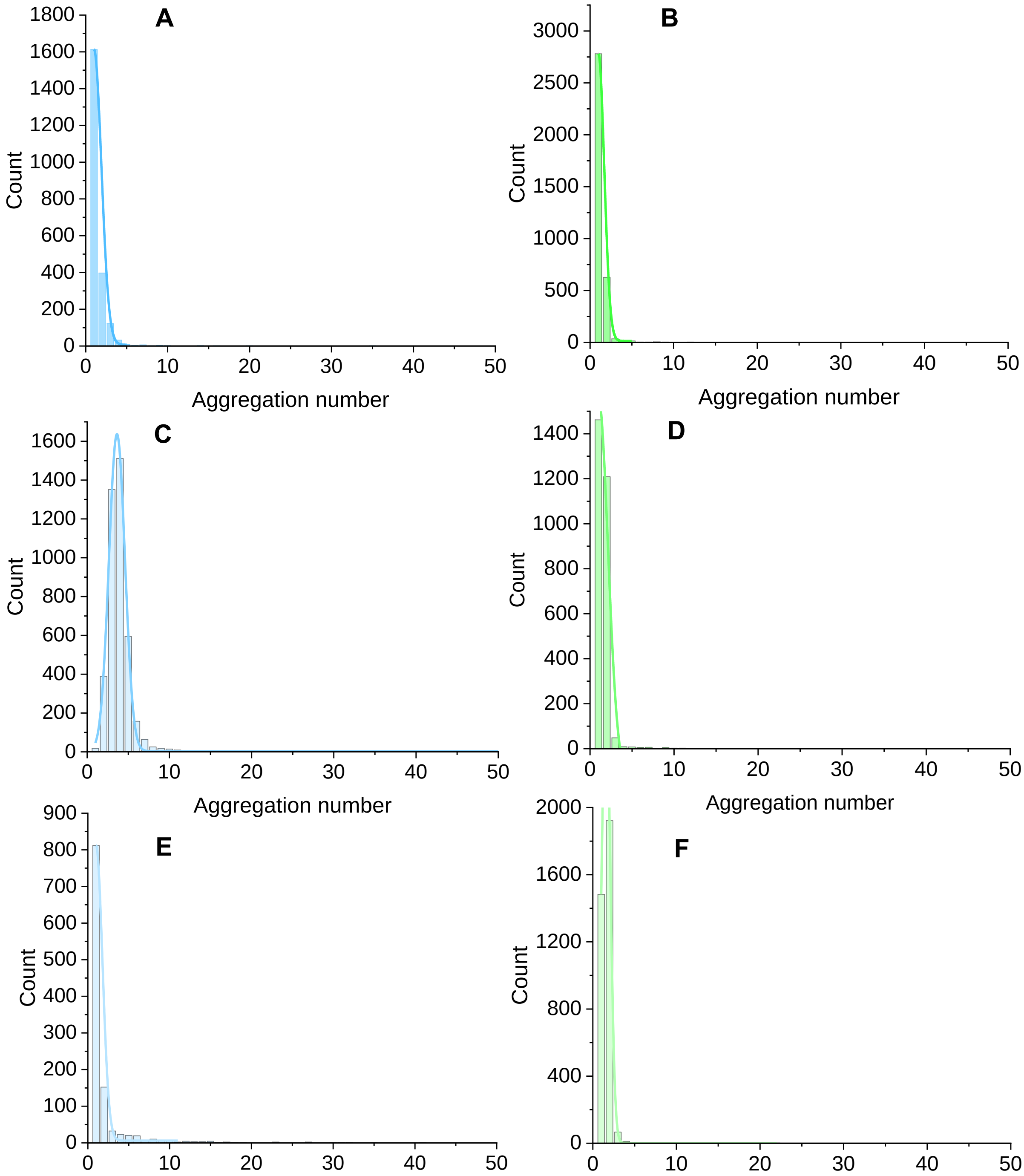

Figure S39. Mass photometry data for all monomeric variants. A-F6A, B-F6S, C-F9A, D-F9S, E-F27A, F-F27S.

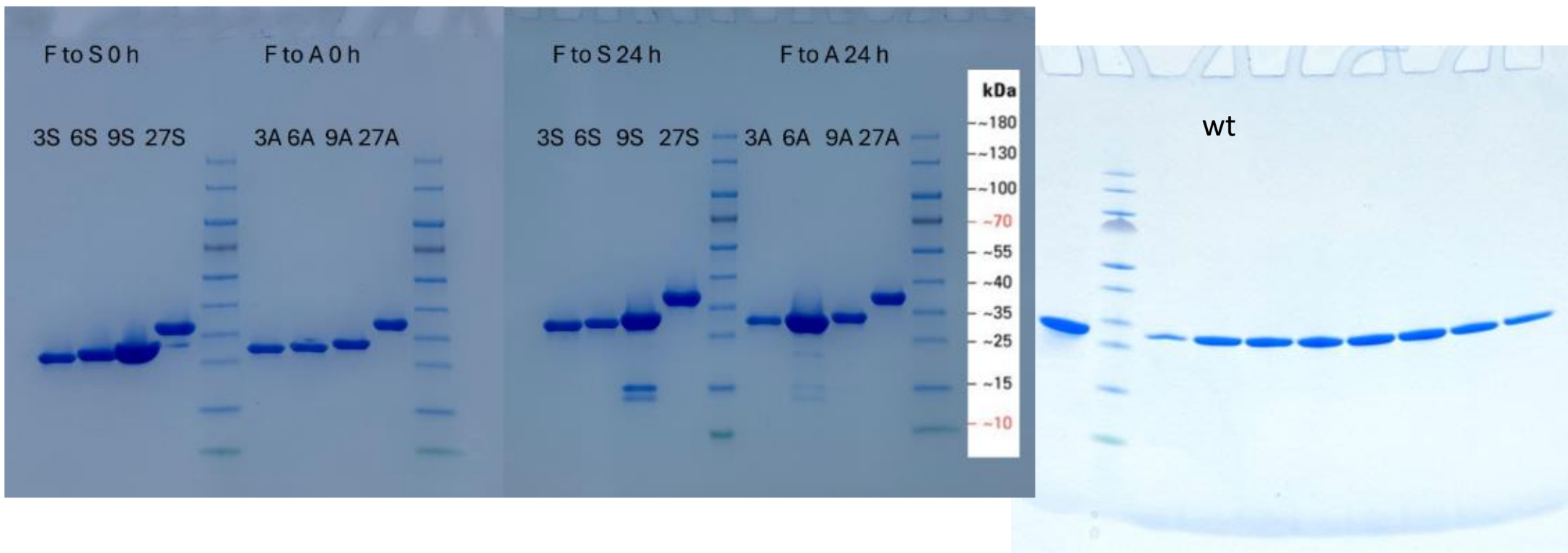

Figure S40. Coomassie-stained 4-20% SDS PAGE gels (Biorad) with tris/glycine as a running buffer of JB6 variant aliquots directly after thawing (left) and after incubation at 37°C.

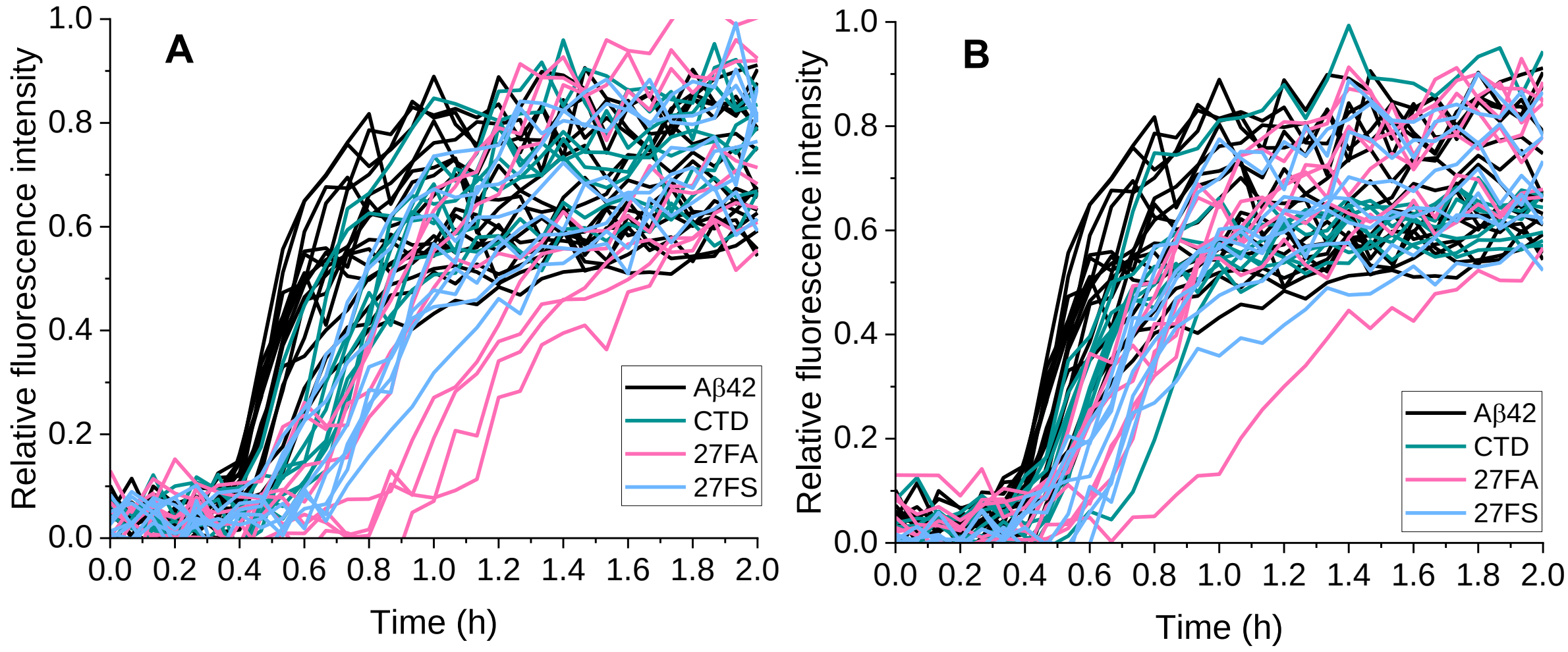

Figure S41. Comparison between Aβ42 aggregation with the presence and absence of the CTD of DNAJB6 and the variants F27S and F27A at 80 nM (A) and 20 nM (B). Aβ42 concentration was 4μM.

References

(1) Österlund, N.; Frankel, R.; Carlsson, A.; Thacker, D.; Karlsson, M.; Matus, V.; Gräslund, A.; Emanuelsson, C.; Linse, S. The C-Terminal Domain of the Antiamyloid Chaperone DNAJB6 Binds to Amyloid-β Peptide Fibrils and Inhibits Secondary Nucleation. J. Biol. Chem. **2023**, 299 (11), 105317. https://doi.org/10.1016/j.jbc.2023.105317.
